# *In Silico* Study of the Anti-MYC Potential of Lanostane-Type Triterpenes

**DOI:** 10.1021/acsomega.4c10201

**Published:** 2024-12-12

**Authors:** José A. C. Oliveira, Jonatas M. Negreiro, Fátima M. Nunes, Francisco G. Barbosa, Jair Mafezoli, Marcos C. Mattos, Maria C. R. Fernandes, Claudia Pessoa, Cristiana L. M. Furtado, Geancarlo Zanatta, Maria C. F. Oliveira

**Affiliations:** a Department of Organic and Inorganic Chemistry, Science Center, Federal University of Ceará, Fortaleza, CE 60455-760, Brazil; b Drug Research and Development Center, Federal University of Ceará, Rua Coronel Nunes de Melo, 1000, Fortaleza, CE 60430-275, Brazil; c Graduate Program in Medical Sciences, University of Fortaleza, Rua Francisco Segundo da Costa, 23-57, Fortaleza, CE 60811-650, Brazil; d Department of Biophysics, Bioscience Institute, Federal University of Rio Grande do Sul, Av. Bento Gonçalves, 9500, Building 43422, Laboratory 204, Porto Alegre, RS 91501-970, Brazil

## Abstract

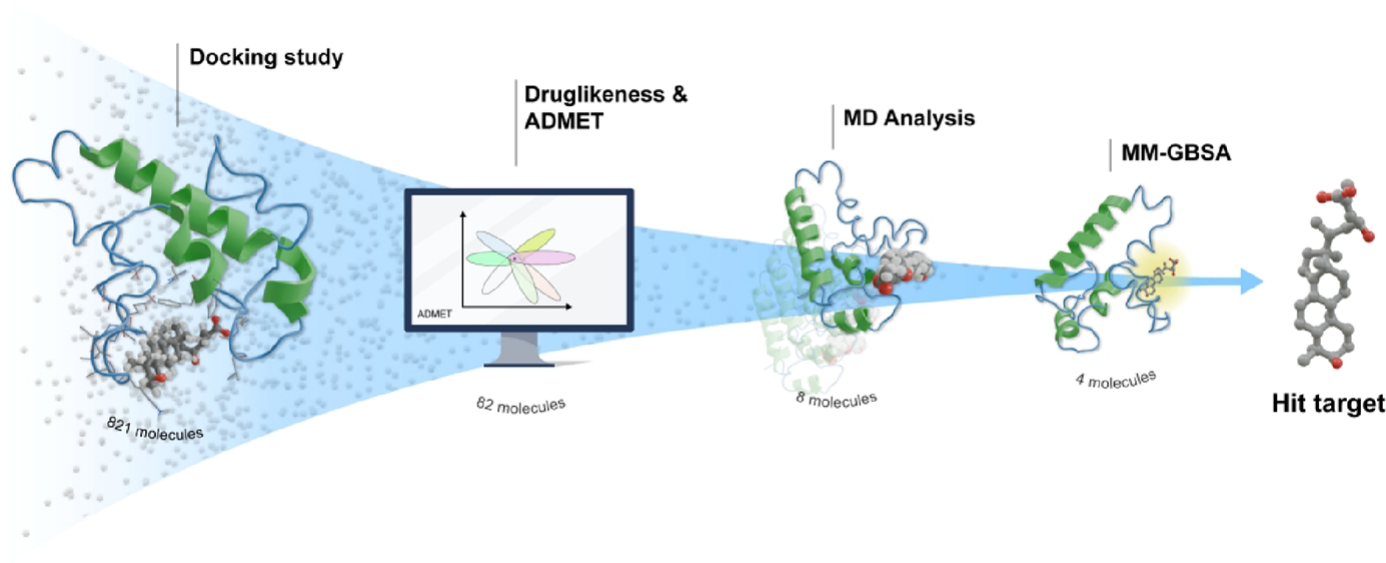

One of the most investigated molecular targets for anticancer therapy is the proto-oncogene *MYC*, which is amplified and thus overexpressed in many types of cancer. Due to its structural characteristics, developing inhibitors for the target has proven to be challenging. In this study, the anti-MYC potential of lanostane-type triterpenes was investigated for the first time, using computational approaches that involved ensemble docking, prediction of structural properties and pharmacokinetic parameters, molecular dynamics (MD), and binding energy calculation using the molecular mechanics-generalized born surface area (MM-GBSA) method. The analysis of physicochemical properties, druglikeness, and pharmacokinetic parameters showed that ligands ganoderic acid E (**I**), ganoderlactone D (**II**), ganoderic acid Y (**III**), ganoderic acid Df (**IV**), lucidenic acid F (**V**), ganoderic acid XL_4_ (**VI**), mariesiic acid A (**VII**), and phellinol E (**VIII**) presented properties within the filter used. These eight ligands, in general, could interact with the molecular target favorably, with interaction energy values between −8.3 and −8.6 kcal mol^–1^. In MD, the results of RMSD, RMSF, radius of gyration, and hydrogen bonds of the complexes revealed that ligands **I**, **IV**, **VI**, and **VII** interacted satisfactorily with the protein during the simulations and assisted in its conformational and energetic stabilization. The binding energy calculation using the MM-GBSA method showed better results for the MYC-**VII** and MYC-**I** complexes (−44.98 and −41.96 kcal mol^–1^, respectively). These results support the hypothesis that such molecules can interact with MYC for a considerable period, which would be an essential condition for them to exert their inhibitory activity effectively.

## Introduction

The MYC (also known as C-MYC) protein is a transcriptional factor belonging to the basic helix–loop–helix zipper (bHLHZiP) family, which plays important roles in regulating gene expression influencing fundamental cellular processes, such as growth, proliferation, differentiation, and cell death.^1−4^ MYC is a proto-oncogene that is highly expressed during embryonic development and becomes inactivated in adult somatic cells.^5,6^ However, oncogenic activation and overexpression of the MYC family are reported in many malignant neoplasms, making it one of the most investigated molecular targets in anticancer therapy.^6−9^ Genomic alterations, epigenetic modifications, and post-translational protein remodeling are associated with MYC activation in human cancers.

MYC overexpression is often associated with gene amplification and chromosomal rearrangements.^10^ Additionally, genetic variants, such as point mutations and indels (insertions/deletions), can enhance MYC protein stability and activity contributing to cancer progression.^11^ Increased enhancer activity or the activation of superenhancers surrounding the MYC locus through mechanisms like chromosomal translocations or retroviral promoter insertion can lead to constitutive activation of MYC expression driving carcinogenesis.^12^ Furthermore, aberrant epigenetic reprogramming, such as the loss of DNA methylation, dysregulated noncoding RNA expression, and changes in histone marks, has been reported to activate the *MYC* proto-oncogene.^13^ Post-translational modification, such as phosphorylation at S62 and *trans*–*cis*-prolyl-isomerization at P63, stabilizes and activates the MYC protein. Arginine methylation of MYC protein has also been suggested to alter its stability.^14^

The *MYC* oncogene has been identified in approximately 75% of all aggressive cancers and is related to low response to available conventional therapies.^5,15^ The mechanisms by which *MYC* is related to cancer development have not yet been fully elucidated. However, it is known that its oncogenic effects occur, in part, through association with MAX (MYC-associated factor X) protein. The heterodimer MYC-MAX binds to promoter regions of target genes involved in cell growth, proliferation, and metabolism contributing to oncogenic effects of MYC.^16−20^ Many challenges are faced in developing MYC inhibitors, as this protein has an intrinsically disordered chemical structure manifesting itself in a dynamic range of unstable conformations devoid of effective sites on its surface.^21^ Due to these characteristics, the molecular target was considered undruggable for a long time.^21−23^ Despite the obstacles, many prototype direct and indirect inhibitors have been designed or are in development.^24−34^ Furthermore, computational methods have also helped the discovery and development of bioactive molecules and enabled the screening of thousands of compounds aiming to identify inhibitors for MYC or MYC-MAX.^35−41^ Until now, only a few small molecules and peptide inhibitors have been reported, and all have demonstrated failures in clinical trials due to their inadequate pharmacokinetic behavior and lack of efficacy under *in vivo* conditions.^40^

**Figure 1:**
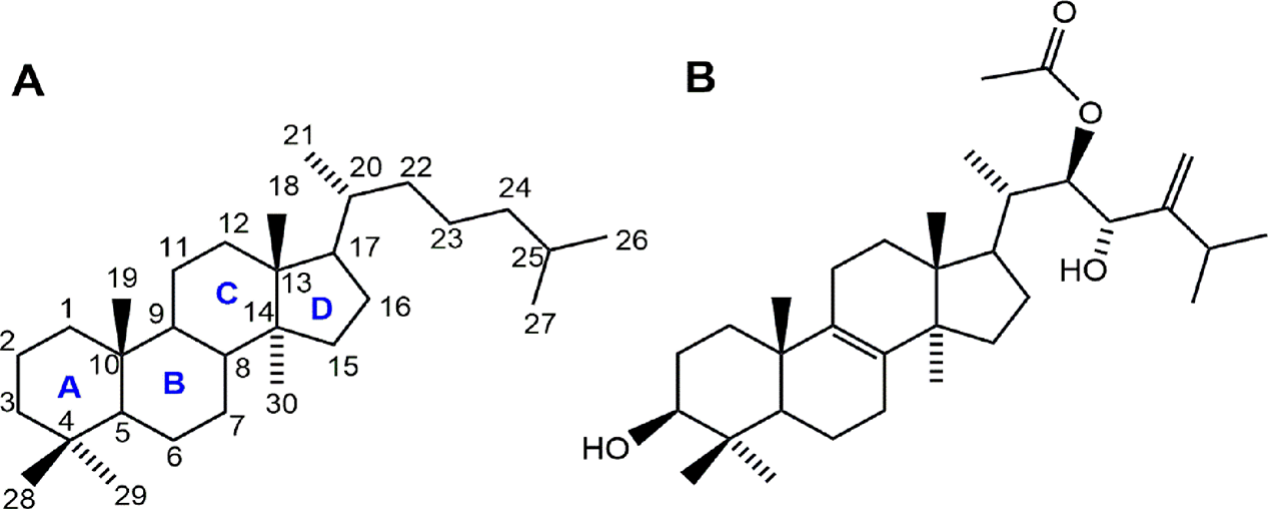
Chemical structures of the lanostane-type triterpene skeleton (A) and pisosterol (B).

In this context, natural products can be investigated as potential bioactive compounds with anti-MYC potential. Among the bioactive natural compounds reported in the literature, lanostane-type triterpenes are molecules biosynthesized by living organisms via the mevalonate pathway.^42^ They are mainly produced by fungi of the genus *Ganoderma* and have diverse structural characteristics that are still being explored.^43,44^ Lanostane triterpenes can have chemical structures with 24, 27, or 30 carbon atoms. The standard skeleton (Figure 1A) is formed by the junction of four rings with trans configurations A/B, B/C, and C/D and may present substituents mainly in positions C-3, C-7, C-11, C-12, C-15, C-22, C-23, C-24, and C-25.^45^ Major related activities include anticancer, anti-HIV, antinociceptive, antimicrobial, anti-AchE, antiviral, antimalarial, and anti-inflammatory.^46−53^

The anticancer potential of pisosterol (Figure 1B), a lanostane-type triterpene produced by the basidiomycete *Pisolithus tinctorius*, has been studied by our group. The cytotoxic effect of this compound was investigated in three animal cell models.^54^ Pisosterol did not show relevant cytotoxicity in mouse erythrocytes or sea urchin embryos but showed selectivity for inhibiting the growth of the tumor cell lines SF-268 (human neuroblastoma), B16 (murine melanoma), PC-3 (human prostate cancer), MCF-7 (human breast cancer), HCT-8 (human colon cancer), HL-60 (human leukemia), and CEM (human leukemia), especially for leukemia and melanoma cells (IC_50_ of 1.55, 1.84, and 1.65 μg mL^–1^ for CEM, HL-60, and B16, respectively).

Another study evaluated the effects of pisosterol on the viability of HL-60 cells over time and at different doses. As a result, a significant drop in cell viability was observed due to the increase in exposure time and concentration of pisosterol, with notable reductions of up to 80% after 72 h at 5.0 μg mL^–1^. The study also highlighted the ability of this triterpene to cause morphological changes in the cytoplasm of these cells.^55^

The *in vivo* study conducted by our research group confirmed the antitumor activity of pisosterol in mice with Sarcoma 180 when they were administered at doses of 50 or 100 mg m^–2^. The percentage of inhibition of tumor growth for the mentioned doses presented rates of 43.0 and 38.7%, respectively. Histopathological analysis to investigate possible toxicity effects showed that the liver and kidney were the main organs affected by pisosterol, although such changes were considered reversible.^56^

Our group also performed a morphological and cytogenetic study using fluorescence *in situ* hybridization analysis of the locus for MYC in two HL-60 cell lines before and after treatment with pisosterol. It was found that at a dose of 1.8 μg mL^–1^, around 15% of the cells showed stained regions, and 39.5% showed few fluorescence signals (3 or 4 alleles), showing that the triterpene probably blocks cells with stained regions in the interphase.^57^ The same behavior was observed for glioblastoma multiforme cells (U343 and AHOL1). Before treatment, 72% of U343 cells and 65% of AHOL1 cells contained more than two *C-MYC* alleles. Pisosterol, when tested at a concentration of 1.8 μg mL^–1^, was able to block gene amplification, as only 33% of U343 cells and 15% of AHOL1 cells showed more than two fluorescence signals.^58^

The antitumor potential of pisosterol, besides the lack of computational studies on the anti-MYC potential of natural products, motivated us to investigate 821 lanostane-type triterpenes as MYC inhibitors using computational methods.

## Results and Discussion

**Figure 2:**
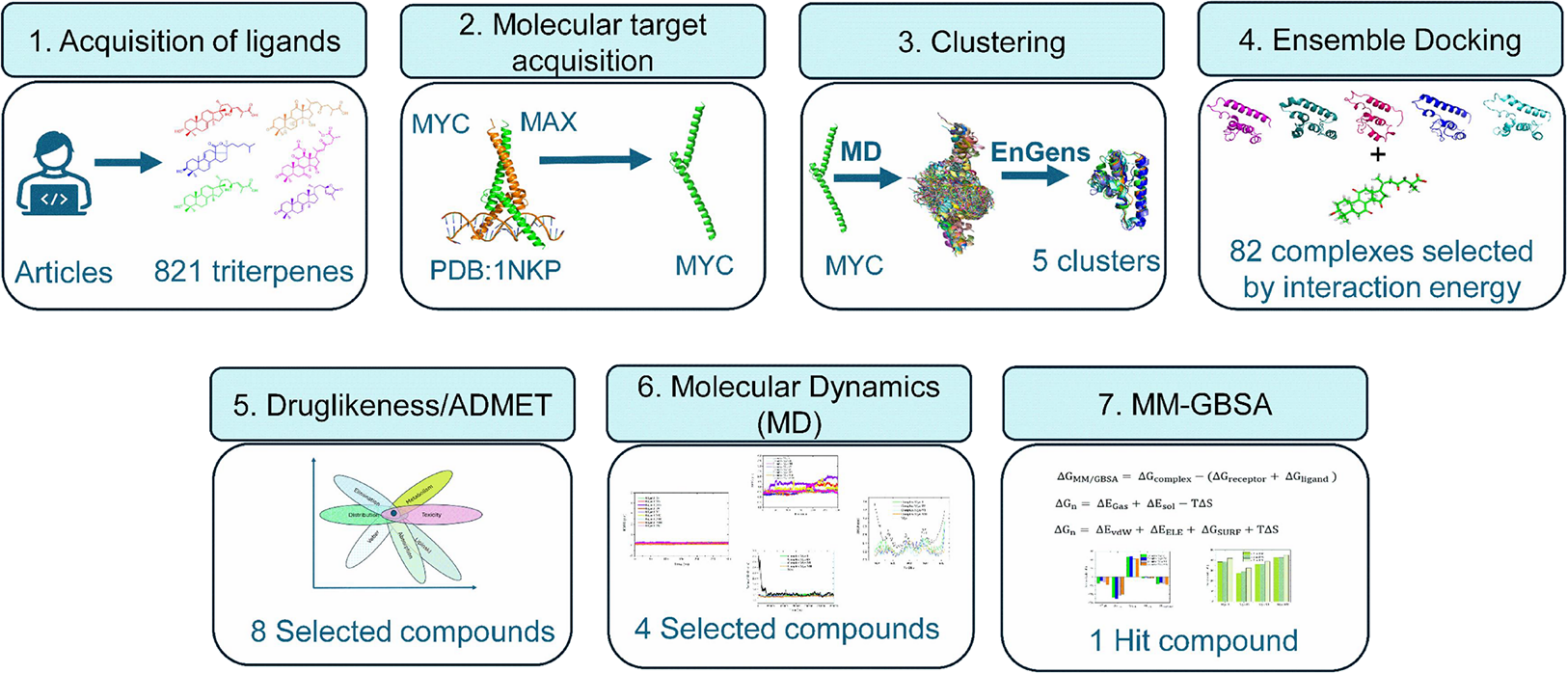
Workflow employed in the study.

The workflow (Figure 2) employed in this study involved structure-based virtual screening using molecular docking studies, prediction of physicochemical (druglikeness) and pharmacokinetic properties, MD simulations, and binding energy calculation using the MM-GBSA method.

### Construction of the Conformational Ensemble

The disordered nature of the MYC protein has been an obstacle to the design of new inhibitors.^38^ Due to the high conformational complexity of this molecular target, it became necessary to build a conformational set of relatively stable structures to improve the chances of ligand coupling. Knowing this, a 400 ns MD simulation of the protein was performed to select relatively stable conformations for ensemble docking studies. Analyses of the RMSD plot and radius of gyration showed that the protein stabilized from 50 to 400 ns (Figure S1, Supporting Information). For the clustering process, the molecular dynamics trajectory was used as an input file; in the dimensionality reduction stage, the PCA method (principal component analysis) and *K*-means were used for the clustering method. Five structural clusters were generated from these analyses, with a silhouette score of 0.008 (Figure S2A, Supporting Information) and clusters with different dimensionalities (Figure S2B, Supporting Information). Finally, the representative conformation of each cluster was used to generate the final conformational set for MYC.

### Ensemble Docking and Virtual Screening

For induced coupling studies, the AutoDock Vina 1.2.0 program was used. The five representative structures of the molecular target, generated by the EnGens tool, were analyzed on the server to identify possible binding sites. The results for predicting each conformation can be seen in Table S1, Supporting Information.

For the structure arising from cluster 0, the binding site with the highest druggability score was identified, which involved the amino acids Gly982, Arg919, Leu943, Lys936, Gly983, Ala946, Gln912, Ile942, Lys918, Thr947, Arg925, Leu931, Val940, Leu924, Ser920, Cys984, Ala937, Phe921, Val941, Lys939, Lys944, Leu917, Glu916, Pro938, Phe922, Asn915, Asn934, Gln980, and Glu935 (Table S1, Supporting Information). Docking simulations of 821 ligands against five conformations of MYC resulted in 41,050 final complexes, which were subsequently ranked according to their interaction energy values. In addition, the interaction energy value of the commercial inhibitor 1074-G5 (−8.3 kcal mol^–1^) was used as a positive control. From the overall results, 10% (82) of the complexes with interaction energy equal to or better than that of the inhibitor were selected (Table S2, Supporting Information). In general, the interaction energy values for these 82 complexes ranged between −8.3 and −10.1 kcal mol^–1^, and 15 complexes had an energy of −8.5 kcal mol^–1^, 12 with −8.4 kcal mol^–1^, and 33 with −8.3 kcal mol^–1^.

### Druglikeness and ADMET Screening

Predicting physicochemical properties and pharmacokinetic parameters *in silico* becomes a fundamental step in developing new active principles.^59^ For these analyses, the stereochemistry reported in the literature was taken into account. The results for evaluating druglikeness and pharmacokinetic properties for the 82 selected ligands can be seen in Tables S3 and S4 (Supporting Information).

Solubility is a property that influences the oral bioavailability of drugs, playing a crucial role in their dissolution in the gastrointestinal environment.^60^ This process represents a determining phase in gastric absorption that precedes the release of the drug into the systemic circulation.^61^ Once present in the body, a substance can be subjected to several metabolization processes where toxic metabolites can be generated. The cytochrome P450 enzymes, present in the liver, for example, are responsible for around 90% of the oxidation of several drugs, and therefore, it is important to predict interactions with their isoforms.^62^ The P-glycoprotein (P-gp) influences the ADMET properties of medicines and toxins; these pumps act as transporters of various compounds out of the cell with energy from ATP. The nonspecificity of substrates of these proteins can reduce the effectiveness of bioactive molecules.^63^ The hERG channel blocking substances, Kv11.1, can cause prolongation of the QT interval, which is associated with the development of cardiotoxicity and an increased risk of cardiac arrest.^64^ Furthermore, in the context of drug discovery, hepatotoxicity is often cited as the main reason for termination of development programs.^65^

Another important aspect in creating new drug prototypes targeted at the central nervous system is the ability of these molecules to overcome the blood–brain membrane. This natural barrier protects the central nervous system and has complicated therapy for brain disorders as most medications have difficulty reaching the brain, resulting in limited therapeutic efficacy and potential side effects on other organs.^66^ Furthermore, compounds of the same class have been effective in the treatment of brain tumors,^67−71^ demonstrating the ability to cross the BBB. Thus, consider that assessing the ability to cross the BBB (>50%) is a fundamental step toward expanding the therapeutic potential of ligands, allowing a broader impact in different areas of oncology.

**Figure 3:**
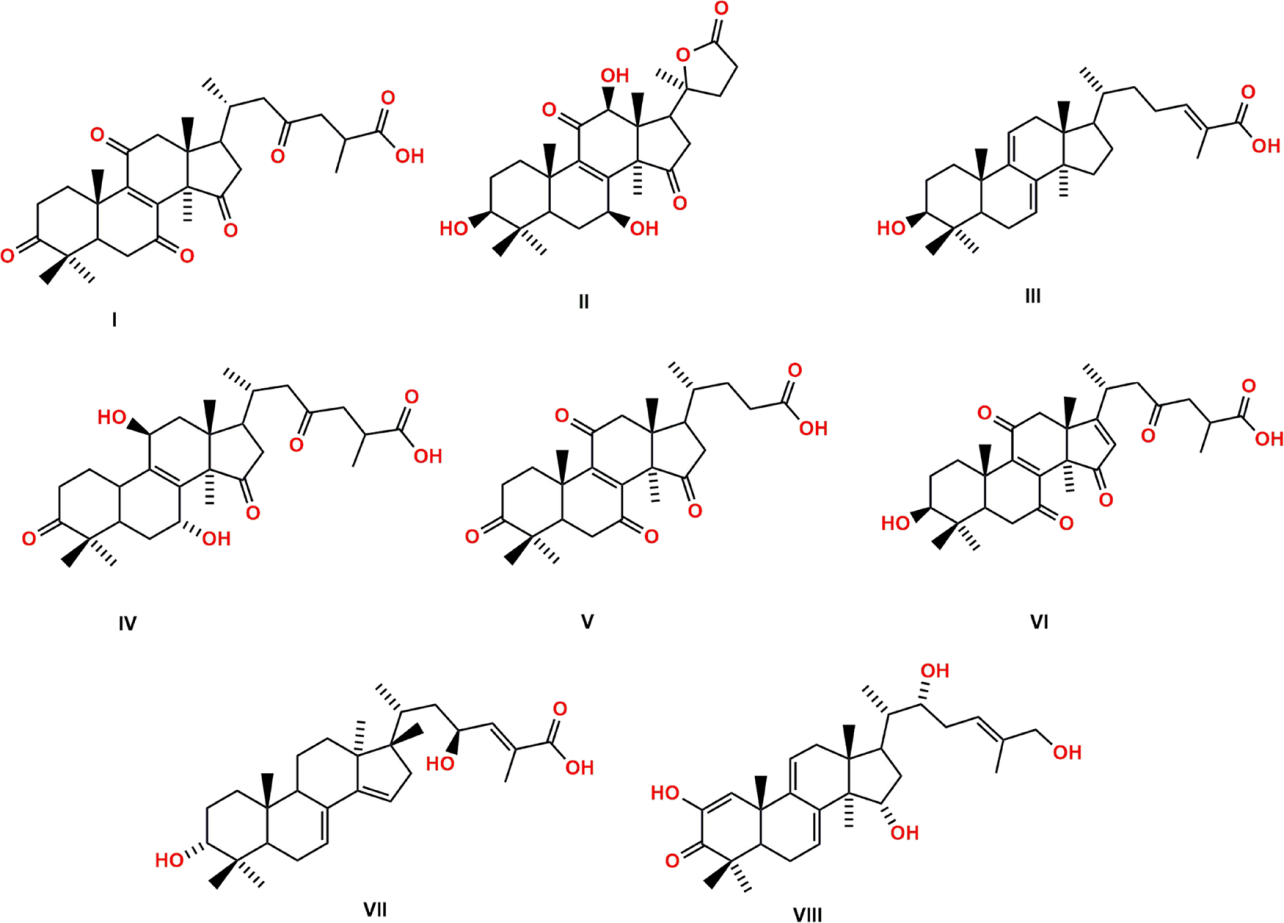
Chemical structures of the lanostane-type triterpene ligands 8-ene-C287 (**I**), 8-ene-C314 (**II**), 7,9-diene-C20 (**III**), 8-ene-C72 (**IV**), 8-ene-C58 (**V**), 8,16-diene-C6 (**VI**), 7,14-diene-C1 (**VII**), and 1,7,9-triene-C4 (**VIII**).

Considering the above aspects, for the selection of the lanostane-type triterpene ligands, the following filter was used: the compound must follow Lipinski and Veber’s rules and present good solubility (Log *D*_7.4_ > 1 < 3), as well as a low probability of being a substrate for P-glycoprotein, GI-A > 50%, BBB > 50%, *T*_1/2_ > 0.5 h, hERG < 50%, and H-HT < 50%. From these analyses, only eight ligands (Figure 3) were selected: 8-ene-C287 (**I**), 8-ene-C314 (**II**), 7,9-diene-C20 (**III**), 8-ene-C72 (**IV**), 8-ene-C58 (**V**), 8,16-diene-C6 (**VI**), 7,14-diene-C1 (**VII**), and 1,7,9-triene -C4 (**VIII**). The physicochemical properties and pharmacokinetic parameters for the eight ligands, selected based on the filter used, can be seen in Tables 1 and 2, respectively.

**Table 1:** Physicochemical Properties for Ligands **I**–**VIII**^a^

ligand	MW (Da)	cLog *P*	HBA	HBD	TPSA (Å^2^)	RB	Log *S*	Log *D*_7.4_
**I**	512.643	4.548	7	1	122.65	6	–5.20	1.30
**II**	474.594	2.482	7	3	121.13	1	–4.43	2.35
**III**	454.695	7.320	3	2	57.53	5	–6.16	2.09
**IV**	502.648	3.741	7	3	128.97	6	–4.97	1.37
**V**	456.579	4.343	6	1	105.58	4	–5.11	1.42
**VI**	512.643	4.260	7	2	125.81	6	–5.05	1.44
**VII**	470.694	6.291	4	3	77.76	5	–5.88	2.04
**VIII**	484.677	5.039	5	4	97.99	5	–5.56	2.11

a Notation: molecular weight (MW), partition coefficient (cLog *P*), hydrogen bond acceptors (HBA), hydrogen bond donors (HBD), topological polar surface area (TPSA), number of rotatable bonds (RB), solubility (Log *S*), and distribution coefficient (Log *D*_7.4_).

**Table 2:** Pharmacokinetic Parameters for the Ligands **I**–**VIII**^a^

ligand	Pgpi (%)	Pgps (%)	HIA (%)	F30 (%)	BBB (%)	*T*_1/2_ (h)	CL (h)	hERG (%)	H-HT (%)
**I**	27.00	3.70	76.30	34.60	95.30	1.72	1.45	37.80	17.40
**II**	58.80	2.80	70.00	45.00	93.90	1.54	1.87	36.50	43.00
**III**	84.10	8.70	80.8	40.90	65.90	1.96	1.16	44.20	48.40
**IV**	38.70	17.50	71.60	46.00	86.00	1.62	1.75	34.50	46.00
**V**	40.90	4.00	74.70	33.30	93.30	1.67	1.57	36.90	29.80
**VI**	41.20	7.00	71.60	31.80	89.80	1.74	1.64	34.70	43.60
**VII**	71.60	11.40	71.70	40.50	35.60	1.94	1.27	38.90	44.60
**VIII**	47.50	34.50	78.90	39.20	22.90	1.69	1.61	38.00	35.80

a Notation: P-glycoprotein inhibitor (Pgpi), P-glycoprotein substrate (Pgps), gastrointestinal absorption (HIA), bioavailability (F30), probability of crossing the blood-brain barrier (BBB), half lifetime (*T*_1/2_), clearance rate (CL), human ether-a-go-go-related gene channel blocker (hERG), and human hepatotoxicity (H-HT).

The selected ligands were triterpenes with carbonyl, hydroxyl, and carboxyl functional groups, that is, highly oxygenated molecules with a strong propensity to act as hydrogen bond donors or acceptors. Most of these triterpenes have acidic groups in their side chains and are related to structural similarities with the presence of unsaturation in their main skeleton. The molecular weight of these ligands varied between 442 and 512 Daltons, presenting a greater lipophilic character, a moderate surface area for the more oxygenated compounds, at least one axis of rotation, and a short but flexible side chain.

Ligand **I**, known as ganoderic acid E, was first isolated from the fungus *Ganoderma lucidum* (*G. lucidum*).^72^ Ligands **II**, **III**, **IV**, and **V**, named ganoderlactone D, ganoderic acid Y, ganoderic acid Df, and lucidenic acid F, respectively, were also isolated from *G. lucidum*. Ligand **II** has already been investigated regarding its potential as an acetylcholinesterase inhibitor.^73^ At the same time, ligand **III** exhibited moderate inhibition of AChE with an IC_50_ of 21.1 ± 2.66 mM,^74^ antiviral activity (20 μg mL^–1^),^75^ the ability to inhibit the enzyme HMG-CoA (IC_50_ = 8.60 μM), and cytotoxicity against the K562 lineage (chronic myeloid leukemia) with an IC_50_ of 17.5 μM.^76^ Ligand **IV** showed potent human aldose reductase inhibitory activity with an IC_50_ of 22.8 μM.^77^ According to the authors of this latter study, the presence of the carboxyl group is important for activity, as its methyl ester has been shown to be much less active. Ligand **V**(78) showed potent inhibitory effects on EBV-EA induction, with IC_50_ values of 352 mol/32 pmol TPA ratio.^79^ Ligand **VI**, known as ganoderic acid XL_4_, was isolated from *Ganoderma theaecolum*,^80^ while ligand **VII** (mariesiic acid A) was isolated from the seeds of *Abies mariesii*, a common plant in Japan.^81^ Ligand **VIII**, known as phellinol E, was isolated from the fungus *Phellinus igniarius* and showed cardioprotective activity against oxygen-glucose deprivation/reoxygenation injury in H9c2 cells at a concentration of 20 μM.^82^

### Analyses of the Complex Interactions

The complexes formed with the eight ligands selected in the screening of the druglikeness and physicochemical parameters were analyzed to understand the molecular behavior of these triterpenes at the MYC site. Table 3 shows the ligands that interacted most effectively with the protein, highlighting the amino acid residues involved in the interactions, the number of hydrogen bonds, bond distance, and the value of the interaction energy of the complexes. Thus, we expect that these ligands may inhibit the formation of the MYC-MAX complex. This type of inhibition may occur through conformational changes in MYC, limiting the binding interface with MAX. Furthermore, these ligands may compete with MAX for shared binding regions on MYC, decreasing the availability of MYC to form the MYC-MAX complex. Small molecules (NSC13728, PKUMDL-YC-1101, PKUMDL-YC-1201, PKUMDL-YC-1202, PKUMDL-YC-1203, PKUMDL-YC-1204, PKUMDL-YC-1301, and L755507)^37,38,40^ identified from computational studies were able to interact with the bHLH-LZ domain of MYC and destabilize the formation of the MYC-MAX complex and reduce its oncogenic activity.^37,38,40^

**Table 3:** Molecular Docking Results for Complexes MYC-**I**–MYC-**VIII**

ligand code	energy (kcal mol^–1^)	H bond	amino acids	distance (Å)
**I**	–8.6	3	Arg919, Ser920, and Phe921	2.69, 2.12, 1.80
**II**	–8.5	3	Leu917, Arg919, and Ser920	1.91, 2.76, 2.92
**III**	–8.4	3	Arg919, Ser920, and Phe921	2.03, 2.21, 2.02
**IV**	–8.3	3	Arg919, Ser920, and Phe921	2.33, 2.16, 1.93
**V**	–8.3	3	Arg919, Ser920, and Phe921	2.09, 2.29, 2.29
**VI**	–8.3	3	Arg919, Ser920, and Phe921	2.33, 2.22, 2.29
**VII**	–8.3	3	Arg919, Ser920, and Phe921	2.10, 2.17, 2.13
**VIII**	–8.3	2	Ser920 and Glu935	2.88, 2.71

The ligands **I**, **III**, **IV**, **V**, **V**, **VI**, and **VII**, due to their structural similarities, formed complexes with MYC through hydrogen bonds involving the amino acids Glu916, Arg919, Ser920, and Phe921 with carboxylate groups present in their side chain (Figure 3); the interaction energy of these complexes varied between −8.3 and −8.6 kcal mol^–1^ (Table 3).

Ligand **VIII** formed a complex with an energy of −8.3 kcal mol^–1^ and made two hydrogen bonds with the amino acids Glu935 and Ser920 with the hydroxyl oxygen present in the side chain and carbonyl present in the C-3 position. Ligand **II** interacted with MYC with an energy of −8.5 kcal mol^–1^ through three hydrogen bonds involving the hydroxyl at C-12 and the carbonyl of the lactone functional group with the amino acids Leu917, Arg919, and Ser920 (Table 3 and Figure S3, Supporting Information).

Considering the absolute value of the interaction energy, the best results were for ligands **I** and **II**. Regarding the interactions involving the amino acids Leu917, Ser920, Phe921, Leu943, and Lys939, it is worth noting that these have already been previously reported for new MYC inhibitor prototypes.^39,83,84^ These amino acids are in the helix–loop–helix region of MYC and are responsible for important interactions for the dimerization process with the MAX protein. When ligands bind to these amino acids, they can interfere with the interaction with the MAX protein, which can impair the formation of the MYC-MAX complex.^39,83,84^ In addition, comparing the interaction energy values of the complexes generated in this study with energy values of complexes formed with ligands described in the literature (D347-2761, 7594-0037, L755507, and 10074-65, Table S2, Supporting Information) and designed for inhibition of MYC or MYC-MAX, we verified a better molecular complementarity in our results (Table S2, Supporting Information).

### Molecular Dynamics Analysis

MD simulations of 300 ns were performed to discern the stability of the best results obtained in the molecular docking study. To analyze the physical movement and to predict conformational changes at the molecular level, calculations of root-mean-square deviation (RMSD), root-mean-square fluctuation (RMSF), radius of gyration (Rdg), and hydrogen bonds for the complexes (MYC-**I**–MYC-**VIII**) were analyzed.

### Analysis of the RMSD of the Complexes

**Figure 4:**
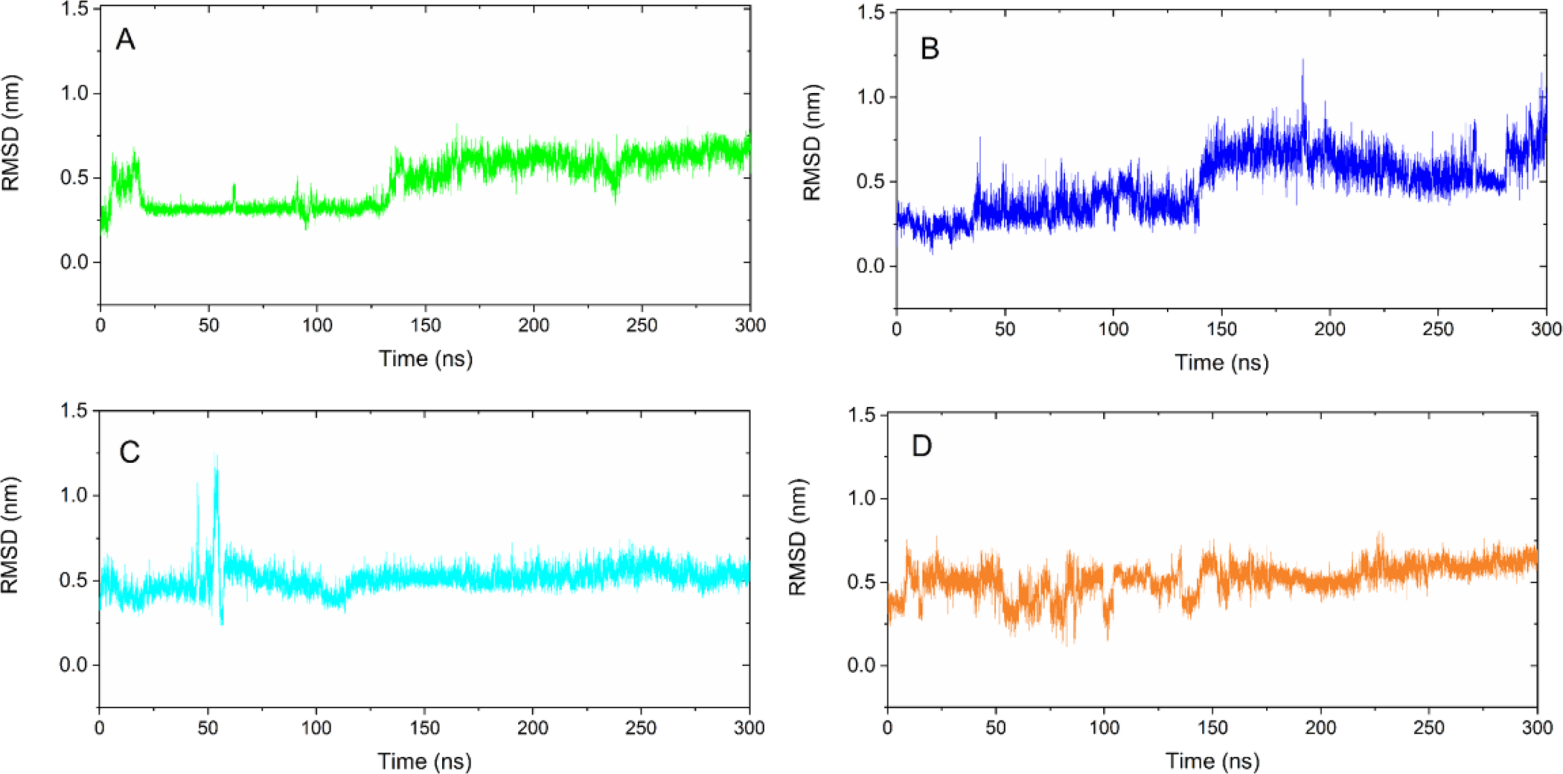
RMSD graph for the complexes. (A) MYC-**I** (green line), (B) MYC-**IV** (blue line), (C) MYC-**VI** (cyan line), and (D) MYC-**VII** (orange line).

The RMSD in molecular dynamics simulations is a measure that evaluates the variability of atomic positions relative to a reference structure.^85^ The RMSD evaluation identifies whether the complex is maintaining its conformational integrity or whether there are significant changes over the simulation time. High RMSD values are associated with more severe conformational changes, while lower values indicate low structural mobility. As observed in the RMSD analysis (Figure S4, Supporting Information), some complexes reached equilibrium at the beginning of the simulation (**I**, **VI**, and **VII**), while others exhibited some fluctuations before equilibrium (**V** and **VIII**) or took most of the simulation to stabilize (**II** and **III**). Overall, RMSD values spanned from 0.48 to 1.34 nm, which reveals relatively low mobility for these systems^86^ (Figure S4, Supporting Information). The complexes formed with ligands **I**, **IV**, **VI**, and **VII** showed greater stability in relation to RMSD values, and their trajectories were analyzed in more detail. The MYC-**I** complex showed stability between 21 and 130 ns, then suffered small fluctuations, and stabilized again from 135 ns until the end of the simulation, with an average RMSD of 0.48 nm (Figure 4, green line). The MYC-I**V** complexes (Figure 4, blue line) showed two stability levels from 40 to 139 ns and 142 to 300 ns; the average RMSD value was 0.48 nm. Analyzing the MYC-**VI** complex (Figure 4, cyan line), we can see a greater stability range that starts from 60 ns and remains practically constant until the end of the simulation; the average RMSD value was 0.51 nm (Figure 4, cyan line). The MYC-**VII** complex showed slight fluctuations in RMSD during the initial 150 ns. It reached relative stability between 160 and 300 ns, with the average RMSD value being 0.52 nm (Figure 4, orange line). For these complexes, it is reinforced that the main conformational changes in the structure of MYC occur mainly in its side chains. The RMSD results for these complexes were promising, as they presented significantly low values with little difference between them. However, considering the absolute mean RMSD value for these complexes, ligands **I**, **IV**, and **VI** were able to interact more effectively with MYC as they showed lower RMSD values. Comparing the results shown in the RMSD graphs of the complexes obtained in this study (Figure 4) with those described,^40,83^ we can verify that the triterpenes used in this study were able to stabilize the MYC structure more quickly and that the average RMSD value was significantly lower, therefore generating more stable complexes. Such behavior may influence the inhibitory potential for these ligands, but to date, a direct relationship between RMSD variation and pharmacological action for this target has not been reported.

### RMSD Analysis for the Ligands at the MYC Binding Site

**Figure 5:**
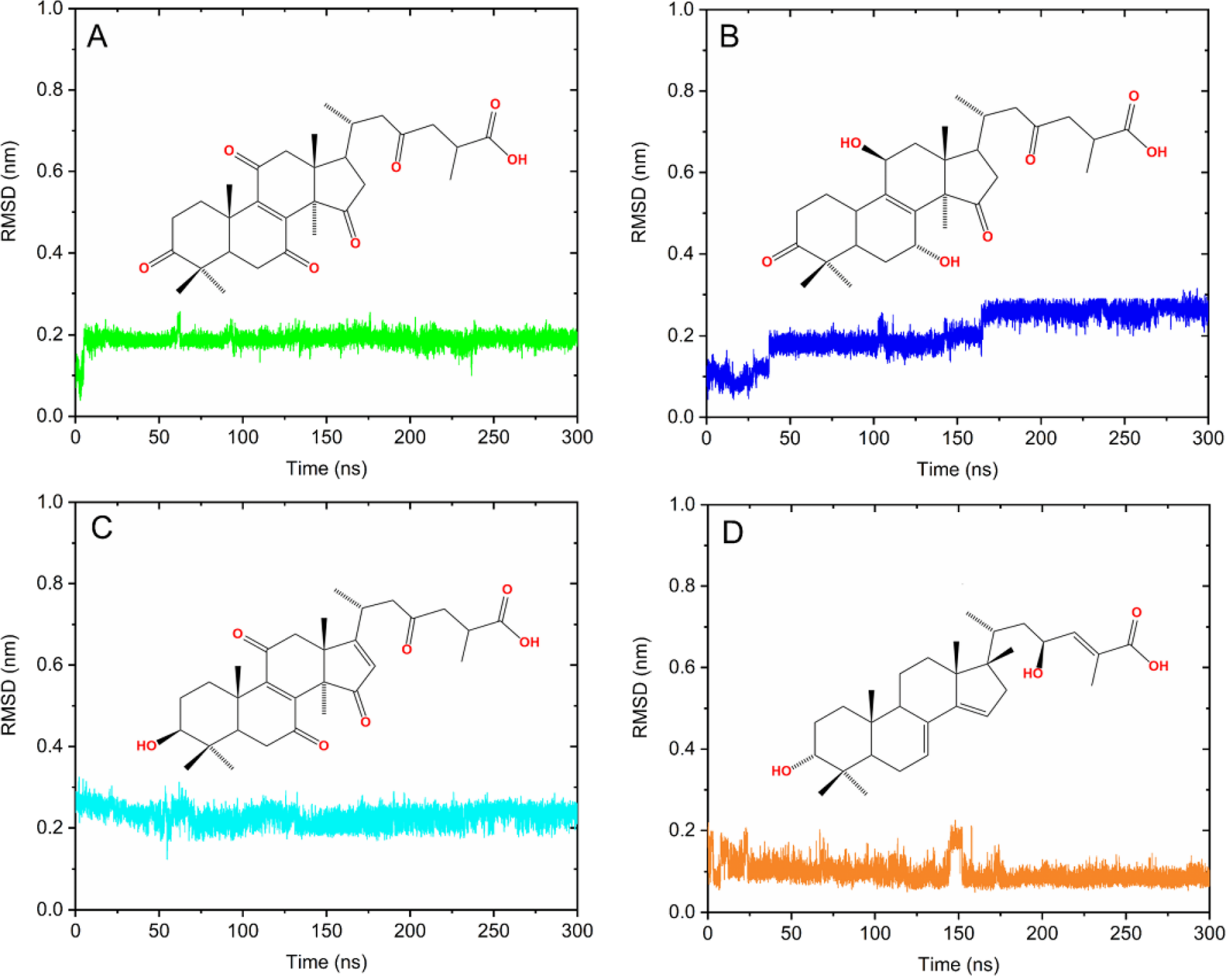
RMSD plot for ligands. (A) Ligand **I** (green line), (B) ligand **IV** (blue line), (C) ligand **VI** (cyan line), and (D) ligand **VII** (orange line).

The molecular behavior of ligands 8-ene-C287 (**I**), 8-ene-C72 (**IV**), 8,16-diene-C6 (**VI**), and 7,14-diene-C1 (**VII**), while interacting with residues at the MYC binding site, was analyzed. The RMSD plot of ligands along 300 ns of simulation is shown in Figure 5. Ligands **IV** and **VI** presented plateaus with RMSD variations and an average value of 0.20 and 0.22 nm, respectively. On the other hand, ligands **I** and **VII** showed a greater stability range throughout the simulation with RMSD values of 0.18 and 0.09 nm, respectively. These results show that the ligands underwent few changes in their conformations when bonded to MYC, reflecting their stabilization at the binding site.

### RMSF Analysis for the Complexes

**Figure 6:**
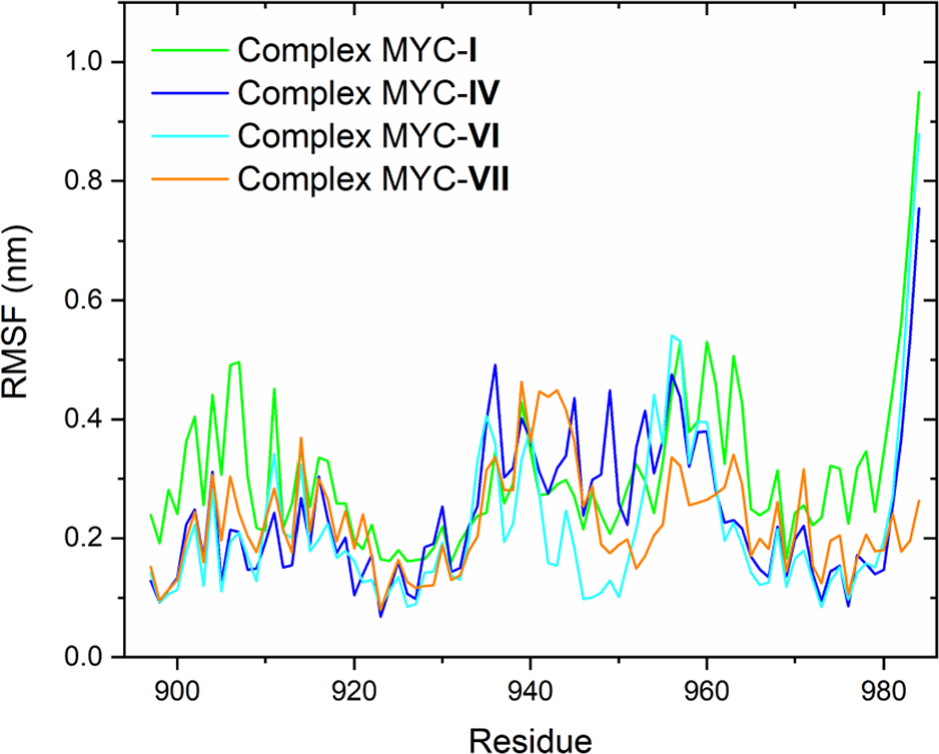
RMSF plot for the complexes MYC-**I** (green line), MYC-**IV** (blue line), MYC-**VI** (cyan line), MYC-**VII** (orange line), and native MYC (black line).

RMSF was used to investigate the flexibility of specific residues of the complexes over time. Discrete fluctuations indicate greater relative stability for the complex. On the other hand, more pronounced fluctuations indicate less stabilization.^87^ The graphs relating to these analyses are shown in Figure 6. More specifically, in the MYC-**IV**, MYC-**VI**, and MYC-**VII** complexes, slight fluctuations were observed in amino acids in regions 906–924 and 965–980, which reflect greater flexibility. Amino acids between 930 and 960, in the central region of the protein, showed more pronounced fluctuations for all complexes (Figure 6). Only the MYC-**I** and MYC-**IV** complexes showed high flexibility when considering the final region. These results show that the presence of the ligands assists in protein stabilization.

### Analysis of the Radius of Gyration of the Complexes

**Figure 7:**
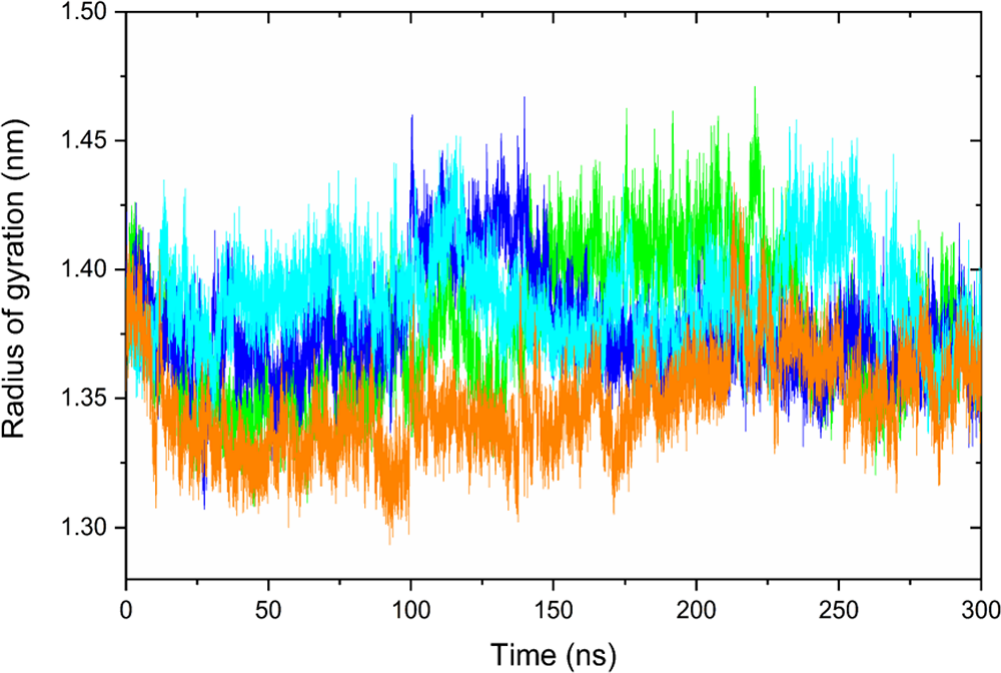
Radius of gyration graph for complexes MYC-**I** (green line), MYC-**IV** (blue line), MYC-**VI** (cyan line), and MYC-**VII** (orange line).

The radius of gyration (Rdg) was used to evaluate the complexes’ size, compactness, and conformational changes throughout the MD simulation. In general, low Rdg values indicate more stable and compact conformations, while higher values are associated with less compact and more expanded structures.^88^ In addition, compaction indicates how molecules are organized and whether they are in conformations that favor effective interactions.^89^ More compact complexes tend to have a configuration that facilitates interaction between components and are therefore more stable, as the interaction between molecules is stronger.^90^ Rdg results are depicted in Figure 7. The complexes MYC-**I**, MYC-**IV**, MYC-**VI**, and MYC-**VII** generally exhibited similar behavior, showing few variations along the trajectory, with values below 1.5 nm.

This shows that, despite the conformational changes in the structure of the MYC protein and ligands throughout the simulation run, the compactness remained practically unchanged.

### Analysis of Hydrogen Bonds during MD

Evaluating the ability of new prototype inhibitors to form hydrogen bonds with specific molecular targets has been a useful strategy for drug screening and design.^91^ The graphs illustrated in Figure S5 (Supporting Information) show that the ligands established interactions with the MYC protein, promoting the formation of hydrogen bonds that help stabilize the complexes. By analyzing the graphs (Figure S5, Supporting Information), ligands **I** and **VII** showed greater oscillations in the number of hydrogen bonds throughout the simulation; for ligand **I**, the number of bonds was between 1 and 4 and for ligand **VII** between 1 and 7 bonds. On the other hand, ligands **IV** and **VI** showed smaller variations in the number of bonds, for ligand **IV** from 4 to 6 bonds on average, and for ligand **VI** between 2 and 4 bonds on average. Thus, the ability of the ligands to carry out stable interactions through hydrogen bonds reinforces the conclusions observed in the RMSD and RMSF, and the radius of gyration analyzes and supports the possibility of these ligands having an anticancer therapeutic effect via inhibition of the MYC protein.

### Analysis of Complex Interactions during MD

**Figure 8:**
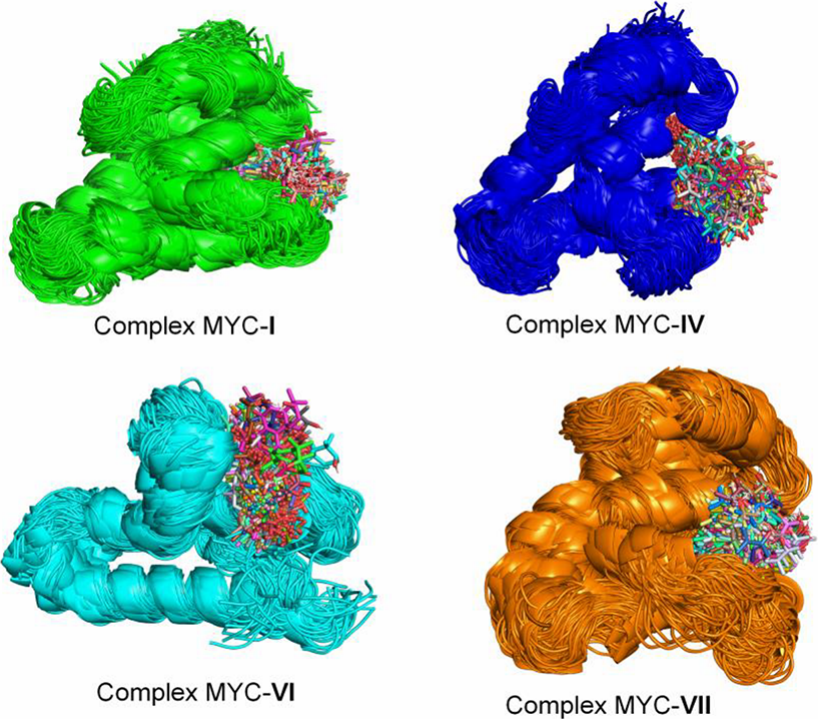
Conformational ensemble of the complexes MYC-**I**, MYC-**IV**, MYC-**VI**, and MYC-**VII** during the 300 ns trajectory.

The integrity of the MYC-**I**, MYC-**IV**, MYC-**VI**, and MYC-**VII** complexes during the 300 ns MD trajectories was verified through analysis in the VMD 1.9.4a51 program.^92^ In general, the ligands remained associated with the MYC binding site, only modifying the types of interactions with the amino acids. Figure 8 shows a summary of the conformations adopted during the simulations for each complex. The ligands investigated formed multiple bonds (hydrophobic and hydrophilic) with the molecular target during the trajectory. These interactions involved the amino acids Leu917, Arg919, Ser920, Phe921, Leu943, Val940, Lys939, Glu916, Ile942, Ala937, Tyr949, Arg925, Pro938, and Leu93, between others (Figures S6–S9, Supporting Information).

### Binding Energy Calculation Analysis Using the MM-GBSA Method

**Figure 9:**
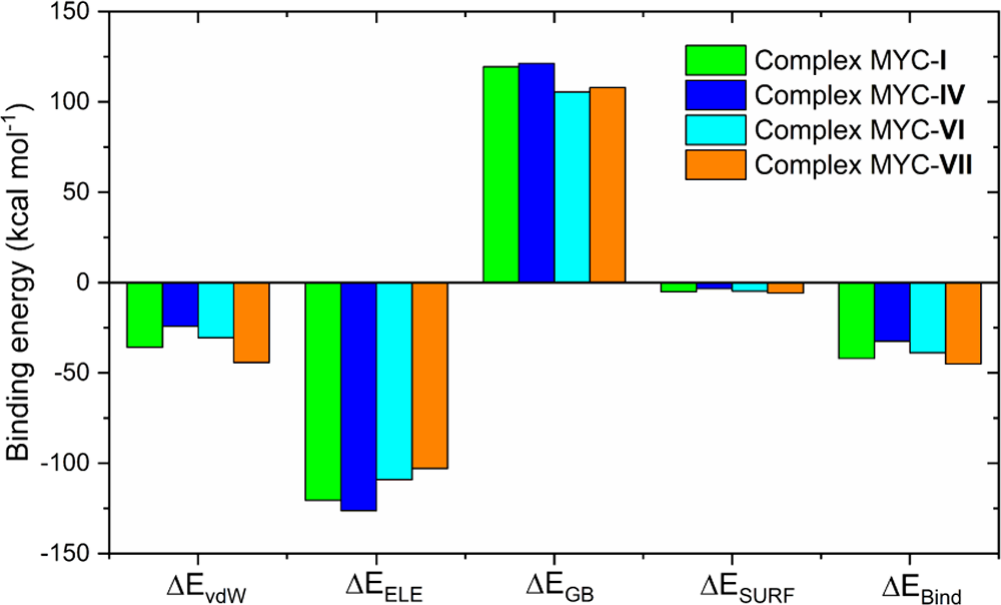
Contributions of the energetic terms associated with the binding energy calculation for the MYC-**I**, MYC-**IV**, MYC-**VI**, and MYC-**VII** complexes.

The MM-GBSA technique is often used to calculate protein–ligand complexes’ final state binding energy.^93^ For the analysis of this study, the last 10 ns (100 frames) of relatively stable trajectories (290–300 ns) was analyzed, at a temperature of 303.15 K. The influence of the external dielectric constant of the solvent on the calculation of binding energy was investigated for values 20, 40, and 80. The binding energy value did not show significant changes for the analyzed constant values (Figure S10, Supporting Information). However, a slightly different response was obtained for the internal solute dielectric constant of 2 and the external solute dielectric constant of 20. The contributions of the energetic terms associated with the (Δ*E*_Bind_) binding energy calculation for the MYC-**I**, MYC-**IV**, MYC-**VI**, and MYC-**VII** complexes are shown in Figure 9. The binding energy decomposition analysis showed positive values for polar solvation energy (Δ*E*_GB_) and negative values for van der Waals interaction energy (Δ*E*_vdW_), electrostatic (Δ*E*_ELE_), and nonpolar contributions (Δ*E*_SURF_). The electrostatic interaction energy values were more significant than the van der Waals interaction energy for final energy (Figure 9). This behavior can be justified by the presence of functional groups (carboxylate) charged on the side chain of the ligands. For the MYC-**I**, MYC-**IV**, MYC-**VI**, and MYC-**VII** complexes, energies of −41.96 ± 3.03, −32.48 ± 3.41, −38.83 ± 2.59, and −44.98 ± 4.41 kcal mol^–1^ were found, respectively (Figure 9). These results are in agreement with the binding energy described for MYC complexes in previous studies.^40,75^ Binding energy values reflect the magnitude of interactions between ligands and proteins, making it possible to estimate the relative stability of these dynamic systems.^94^ In this case, the interactions of the molecular target MYC with the ligands 7,14-diene-C1 (**VII**) proved to be energetically more favorable compared to the other ligands. Such observations suggest strong connections in the complexes, which can be translated as greater inhibitory action and, consequently, greater potential for therapeutic effects.

**Figure 10:**
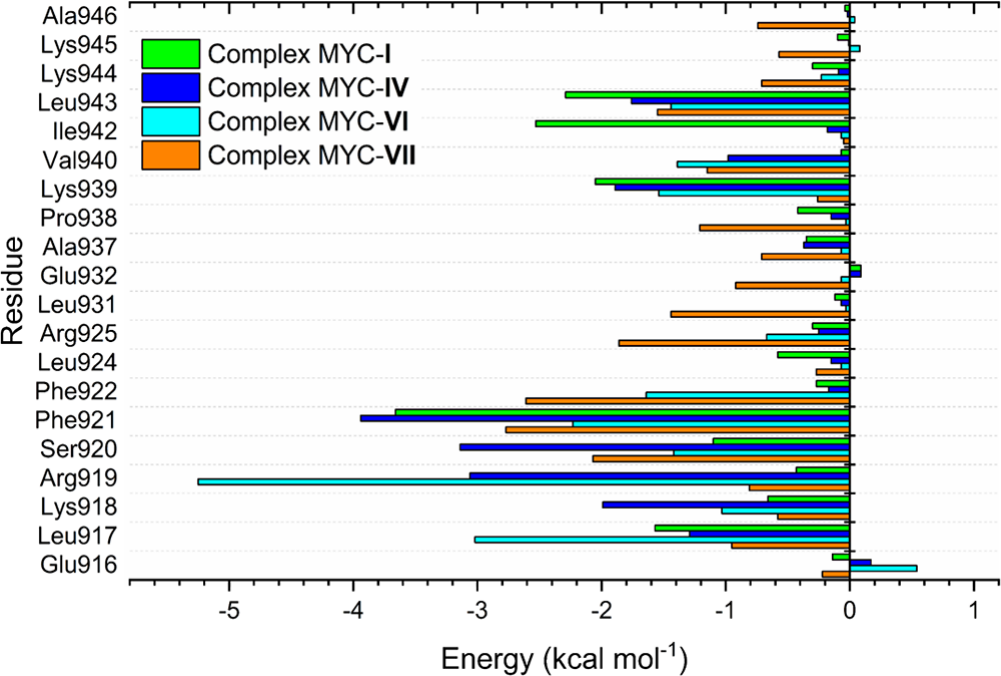
Contributions of the energetic terms associated with the binding energy calculation for the MYC-**I**, MYC-**IV**, MYC-**VI**, and MYC-**VII** complexes.

The contribution of the main amino acid residues to the energy of binding was investigated, covering amino acids distant up to 10 Å from the ligands (Figure 10). The most favorable contributions were identified in the four complexes (MYC-**I**, MYC-**IV**, MYC-**VI**, and MYC-**VII**), and the complexes exhibited distinct energetic decompositions for the binding energy. In the MYC-**I** complex, the main contributions were Phe921 (Δ*E* = −3.66 kcal mol^–1^), Lys939 (Δ*E* = −2.05 kcal mol^–1^), Ile942 (Δ*E* = −2.53 kcal mol^–1^), and Leu943 (Δ*E* = −2.29 kcal mol^–1^) (Figure 10). In the MYC-**IV** complex, the main contributions were Arg919 (Δ*E* = −3.06 kcal mol^–1^), Ser920 (Δ*E* = −3.14 kcal mol^–1^), and Phe921 (Δ*E* = −3.94 kcal mol^–1^) (Figure 10). For the MYC-**VI** complex, the contributions of amino acid residues Leu917 (Δ*E* = −3.02 kcal mol^–1^), Arg919 (Δ*E* = −5.25 kcal mol^–1^), and Phe921 (Δ*E* = −2.18 kcal mol^–11^) (Figure 10) were obtained. Completion analysis of the MYC-**VII** complex showed that amino acids developed more for binding energy binding of Ser920 (Δ*E* = −2.07 kcal mol^–1^), Phe921 (Δ*E* = −2.77 kcal mol^–1^), and Phe922 (Δ*E* = −2.61 kcal mol^–1^) (Figure 10). The amino acids Leu917, Ser920, and Phe921 showed strong contributions to binding energy in previous studies, and as hot spots, the amino acids Leu924, Gln927, and Leu943 stood out.^40^

Based on the computational studies carried out so far, the 7,14-diene-C1 (**VII**) ligand stands out as the hit compound for MYC inhibition. This triterpene showed an interaction energy of −8.3 kcal mol^–1^, follows those of Lipinski and Veber, has ADMET parameters within the filter used, and showed strong interaction with a molecular target in molecular dynamics simulation studies, justified through RMSD, RMSF, Rdg, and hydrogen bonds analyses. The complex formed with this triterpene showed the best binding energy with a value of −42.64 kcal mol^–1^. A triterpene belonging to the class of compounds called pisosterol has shown anticancer potential^54−56,95^ and the ability to act in the attenuation of cancer cell lines with MYC overexpression.^57,58^ Therefore, all these results are important and could represent a breakthrough for developing new agents of anticancer and MYC inhibitors.

## Methods

### Ligand Library and Preparation

The lanostane-type triterpene compounds were selected from articles published in the literature between 1956 and 2022. The 2D structures of the compounds (ligands) were generated using ChemDraw Ultra 12.0 software.^96^ Avogadro software (version 1.2) was employed to produce 3D conformations and to perform energy minimization procedures using the universal force field (UFF).^97^ When necessary, Open Babel (version 2.3.1) was employed to convert 3D structures into pdbqt format.^98^ The (*R*) or (*S*) stereochemistry of each lanostane-type triterpene structure was represented as indicated in the original literature.

### Molecular Target Selection and Preparation

The three-dimensional structure of the MYC-MAX heterodimer complexed to the DNA molecule was obtained from the Protein Data Bank (PDB)^99^ with the ID 1NKP.^100^ In PyMOL v2.5.3 software,^101^ MYC was separated from the MAX protein and DNA molecule, and the water molecules were removed. The protonation step at pH 7.4 was performed using the PROPKA algorithm, available on the PDB2PQR server (https://server.poissonboltzmann.org/pdb2pqr),^102^ and the stereochemical quality of the protein was evaluated by the Ramachandran plot generated from the PROCHECK server (https://saves.mbi.ucla.edu/).^103^

### Conformational Ensemble

Initially, using the GROMACS v2023 program,^104^ a 400 ns MD simulation of the protein in water was carried out to obtain relatively stable conformations during the trajectory. To carry out the simulation, the traditional protocol was used, which consists of the steps of preparing the topology files, defining the box and solvent, adding ions, minimizing energy, equilibration, and production. From the MD trajectory, the EnGens tool was used to build the representative conformational set of the molecular target, according to the methodology described by Conev and co-workers (https://github.com/KavrakiLab/EnGens).^105^

### Determination of the Binding Site

The CavityPlus web server was used to evaluate possible binding sites in the protein’s three-dimensional structure (http://www.pkumdl.cn:8000/cavityplus/index.php).^106^ This tool identifies pockets in three-dimensional protein structures and classifies them with drug scores and druggability scores. The search for binding sites was carried out using the five conformations generated by the EnGens tool. The binding site was selected based on the druggability score and similarity with previous studies.^40,84^

### Ensemble Docking

Molecular docking simulations were performed using AutoDock Vina 1.2.0.^107^ Five three-dimensional structures of the molecular target generated by the EnGens tool were used, with a simulation box centered at 58.75, 63.75, and 52.50 for the *X*, *Y*, and *Z* coordinates, respectively, and a size of 15 × 12 × 14 Å.

### Druglikeness Prediction and Pharmacokinetic Parameters

The SwissADME webserver (http://www.swissadme.ch/)^108^ was used to estimate the druglikeness, distribution coefficient (Log *D*_7.4_), and aqueous solubility (Log *S*) of the ligands. In addition, the Lipinski and Veber rules were used for screening through oral bioavailability.^109,110^ To evaluate these rules, the molecular weight (MW), number of hydrogen bond donor (HBD) and acceptor (HBA) atoms, partition coefficient (cLog *P*), topological polar surface area (TPSA), and number of rotatable bonds (RB) were analyzed. The evaluation of the absorption, distribution, metabolism, elimination, and toxicity (ADMET) parameters of the lanostane-type triterpenes was carried out using the ADMETlab platform (https://admet.scbdd.com/home/index/).^111^ The properties analyzed for screening were the P-glycoprotein inhibitor (Pgpi), P-glycoprotein substrate (Pgps), gastrointestinal absorption (HIA), bioavailability (F30), probability of crossing the blood–brain barrier (BBB), half lifetime (*T*_1/2_), clearance rate (CL), human ether-a-go-go-related gene (hERG) channel blocker, and human hepatotoxicity (H-HT).

### Molecular Dynamics Simulations

Molecular dynamics (MD) simulations were conducted using GROMACS 2023 software.^104,112^ For the protein under study, the topology parameters were defined with the aid of the AMBER99SB force field.^113^ The topology parameters for the ligands were generated using the ACPYPE (AnteChamber Python Parser interfacE) server (https://www.bio2byte.be/acpype/).^114,115^ The initial conformation of the ligands used for each complex was the best pose, selected from induced coupling studies carried out using the ensemble docking technique. Each complex was solvated in a cubic box with tip3p water, with periodic boundary conditions applied, and the system neutralized with Na^+^ or Cl^–^ ions (0.15 mol L^–1^). Before the simulation, each complex underwent energy minimization, which consisted of 10,000 steps of the steepest descent algorithm followed by a 10,000-step conjugate gradient algorithm, thus ensuring system stability. Subsequently, each molecular model was gradually heated from 0 to 303.15 K in the isothermal isovolumetric (NVT) ensemble for 200 ps. It was then equilibrated for 1.0 ns in the isothermal–isobaric set (NPT) at 303.15 K and 1.0 atm.

### Calculation of Binding Energy (MM-GBSA)

The gmx_MMPBSA 1.6.3 package, an efficient GROMACS tool, was used to calculate binding energy using the MM-GBSA method (https://valdes-tresanco-ms.github.io/gmx_MMPBSA/dev/).^116^ To calculate the binding energy, the last 10 ns of relative stability (100 frames) of the complex’s trajectory (290–300 ns) were used, with an internal dielectric constant of 2, external dielectric constants of 20, 40, 80, and a temperature of 303.15 K. The contribution of the total energy components Δ*G*_Bind_ is described in eqs 1–3below, where Δ*G*_complex_, Δ*G*_receptor_, and Δ*G*_ligand_ represent the estimated binding energy of the complex, protein, and ligand, respectively. Δ*G*_*n*_ refers to the contribution of each individual entity (complex, protein, and ligand). The term Δ*E*_Gas_ represents the variation in interaction energy between the protein and the ligand in the gas phase, obtained through van der Waals (Δ*E*_vdW_) and electrostatic (Δ*E*_ELE_) interactions. Meanwhile, the term Δ*E*_sol_ reflects the binding energy of solvation, derived by calculating the polar (Δ*E*_GB_) and nonpolar contributions. Finally, *T*Δ*S* corresponds to the entropy term but was not considered in the calculations.
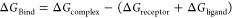
1
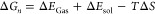
2
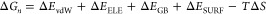
3

## Conclusions

The anti-MYC potential of triterpenes was evaluated using computational approaches involving molecular docking, MD, and binding energy calculation. Using the EnGens tool, the relatively stable MYC protein conformational ensemble was generated to track the initial binding poses of the triterpenes. In the docking studies, 82 ligands were selected based on the interaction energy values between −8.3 and −10.1 kcal mol^–1^. The virtual screening of these 82 triterpenes using physicochemical (druglikeness) and pharmacokinetic (ADMET) properties allowed the selection of eight triterpenes. The stability of these eight complexes was analyzed by 300 ns MD simulations. The RMSD, RMSF, and radius of gyration plots revealed that four ligands, 8-ene-C287 (**I**), 8-ene-C72 (I**V**), 8,16-diene-C6 (**VI**), and 7,14-diene-C1 (**VII**), effectively interacted with the protein during the simulation and helped its stabilization. The binding energy was calculated using the MM-GBSA method and presented energies between −32.48 and −44.98 kcal mol^–1^ for the complexes MYC-**I**, MYC-**IV**, MYC-**VI**, and MYC-**VII**.

The computational results obtained in this study support the hypothesis of strong interactions between the molecular target and the lanostane-type ligands, especially for the 7,14-diene-C1 (**VII**) ligand, which was considered a hit compound. This behavior may suggest a possible inhibitory action for these molecules, motivating further *in vitro* anti-MYC experiments.

## Data Availability

To construct the conformational set of the MYC protein, the EnGens pipeline provided on GitHub (https://github.com/KavrakiLab/EnGens/blob/main/README.md) was used. Ensemble docking studies were carried out using the free AutoDock Vina v1.2.0 program. MD simulations were conducted in GROMACS 2023. The energy calculation using the MM-GBSA method was performed using the gmx_MMPBGBSA 1.6.3 package available on GitHub (https://valdes-tresanco-ms.github.io/gmx_MMPBSA/dev/). The input files and main results can be located in Zenodo (10.5281/zenodo.11226836).

## Supporting Information Available

MD for MYC protein; MD trajectory clustering of MYC protein obtained by the EnGens pipeline; molecular docking poses for the top eight complexes; RMSD plots for the top eight complexes; hydrogen bonding plots for the complexes with ligands **I**, **IV**, **VI**, and **VII**; interactions observed in MYC-**I**, MYC-**IV**, MYC-**VI**, and MYC-**VII** complexes during the 300 ns MD trajectory; influence of the solvent dielectric constant on interaction energy calculation; binding sites identified by the CavityPlus server; interaction energy results for the 82 ligands; druglikeness, physicochemical, and pharmacokinetic properties for the 82 selected ligands (PDF)
