# Supplementary material for: In Silico Study of the Anti-MYC Potential of Lanostane-Type Triterpenes
Source: ACS Omega. 2024 Dec 12;9(51):50844–58. doi: 10.1021/acsomega.4c10201 (PMC11683602; doi:10.1021/acsomega.4c10201)
Supplement: Supplementary file 1 — ao4c10201_si_001.pdf [file ao4c10201_si_001.pdf]

In Silico Study of the Anti-MYC Potential of Lanostane-type Triterpenes

José A. C. Oliveira,[a] Jonatas M. Negreiro,[a] Fátima M. Nunes,[a] Francisco G. Barbosa,[a] Jair Mafezoli,[a] Marcos C. Mattos,[a] Maria C. R. Fernandes,[b] Claudia Pessoa,[b] Cristiana L. M. Furtado,[b,c] Geancarlo Zanatta,[d,] Maria C. F. Oliveira [a,*]*

[a]Department of Organic and Inorganic Chemistry, Science Center, Federal University of Ceará, Fortaleza-CE, 60455-760, Brazil.

[b]Drug Research and Development Center, Federal University of Ceará. Rua Coronel Nunes de Melo, 1000, Fortaleza-CE, 60430-275, Brazil.

[c]Graduate Program in Medical Sciences, University of Fortaleza, Rua Francisco Segundo da Costa, 23-57, Fortaleza-CE, 60811-650, Brazil.

[d]Department of Biophysics, Bioscience Institute, Federal University of Rio Grande do Sul, Av. Bento Gonçalves, 9500, Building 43422, Laboratory 204, Porto Alegre-RS, 91501-970, Brazil.

SUPPORTING INFORMATION

* Corresponding authors: G. Zanatta (geancarlo.zanatta@ufrgs.br) and M. C. F. Oliveira (mcfo@ufc.br)

Table of Contents

**Figure S1.**	(A) RMSD plot for MYC protein, (B) graph of the radius of gyration for the MD trajectory of the MYC protein.	S4
**Figure S2.**	(A) Clusters for MYC generated by the EnGens tool, (B) Molecular weight associated with structural clusters generated by the EnGens tool.	S5
**Figure S3.**	Result for the best molecular docking poses for the complexes MYC-**I** (green), MYC-**II** (red), MYC-**III** (violet), MYC-**IV** (blue), MYC-**V** (yellow), MYC-**VI** (cyan), MYC-**VII** (orange), and MYC-**VIII** (magent).	S6
**Figure S4.**	RMSD graph for the complexes MYC-**I** (green line), MYC-**II** (red line), MYC-**III** (violet line), MYC-**IV** (blue line), MYC-**V** (yellow line), MYC-**VI** (cyan line), MYC-**VII** (orange line), and MYC-**VIII** (magent line).	S7
**Figure S5.**	Hydrogen bond plots for the complex. (A) MYC-**I** (green line), (B) MYC-**IV** (blue line), (C) MYC-**VI** (cyan line), (D) MYC-**VII** (orange line).	S8
**Figure S6.**	Interactions observed for the MYC-**I** complex during an MD trajectory at 50 ns, 100 ns, 150 ns, 200 ns, 250 ns and 300 ns.	S9
**Figure S7.**	Interactions observed for the MYC-**IV** complex during an MD trajectory at 50 ns, 100 ns, 150 ns, 200 ns, 250 ns, and 300 ns.	S10

**Figure S8.**	Interactions observed for the MYC-**VI** complex during an MD trajectory at 50 ns, 100 ns, 150 ns, 200 ns, 250 ns, and 300 ns.	S11
**Figure S9.**	Interactions observed for the MYC-**VII** complex during an MD trajectory at 50 ns, 100 ns, 150 ns, 200 ns, 250 ns, and 300 ns.	S12
**Figure S10.**	Influence of the external dielectric constant of the solvent on the calculation of free energy for values 20, 40, and 80.	S13
**Table S1.**	Binding site identified by the cavityplus server for clusters 0-4.	S14
**Table S2.**	Interaction energy results for the 82 ligands selected by induced coupling and inhibitors described in the literature.	S15
**Table S3.**	Druglikeness and physiochemical properties for the 82 selected ligands.	S32
**Table S4.**	Pharmacokinetic parameters for the 82 ligands selected by induced coupling.	S35
**REFERENCES**	List of references cited	S37

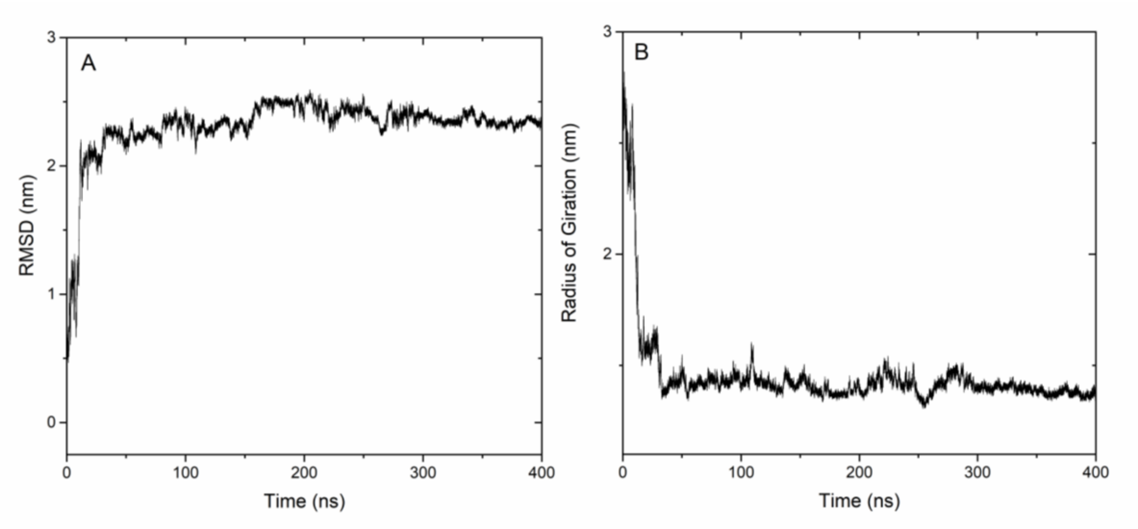

Figure S1. (A) RMSD plot for MYC protein. (B) graph of the radius of gyration for the molecular dynamics trajectory of the MYC protein.

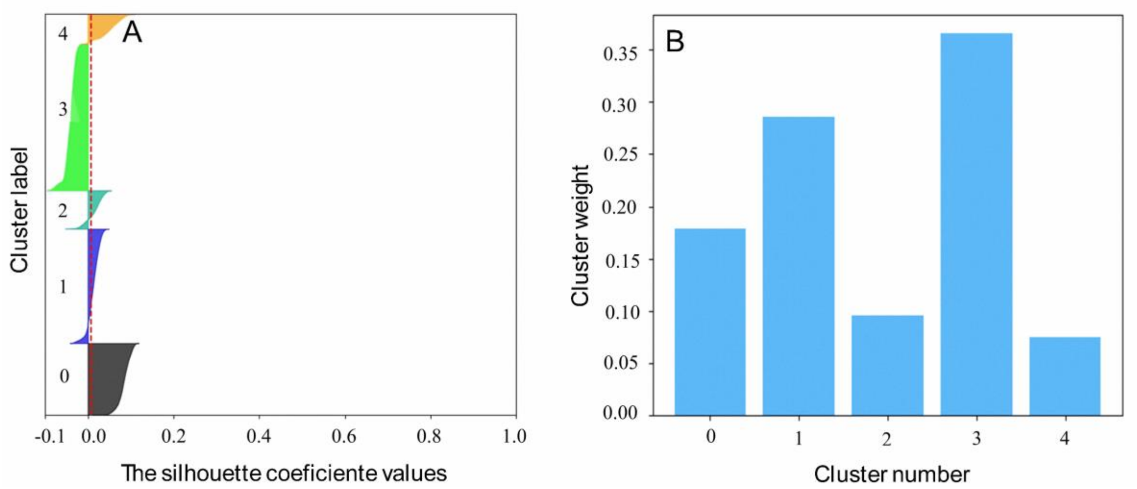

Figure S2. (A) Clusters for MYC generated by the EnGens tool. (B) Molecular weight associated with structural clusters generated by the EnGens tool.

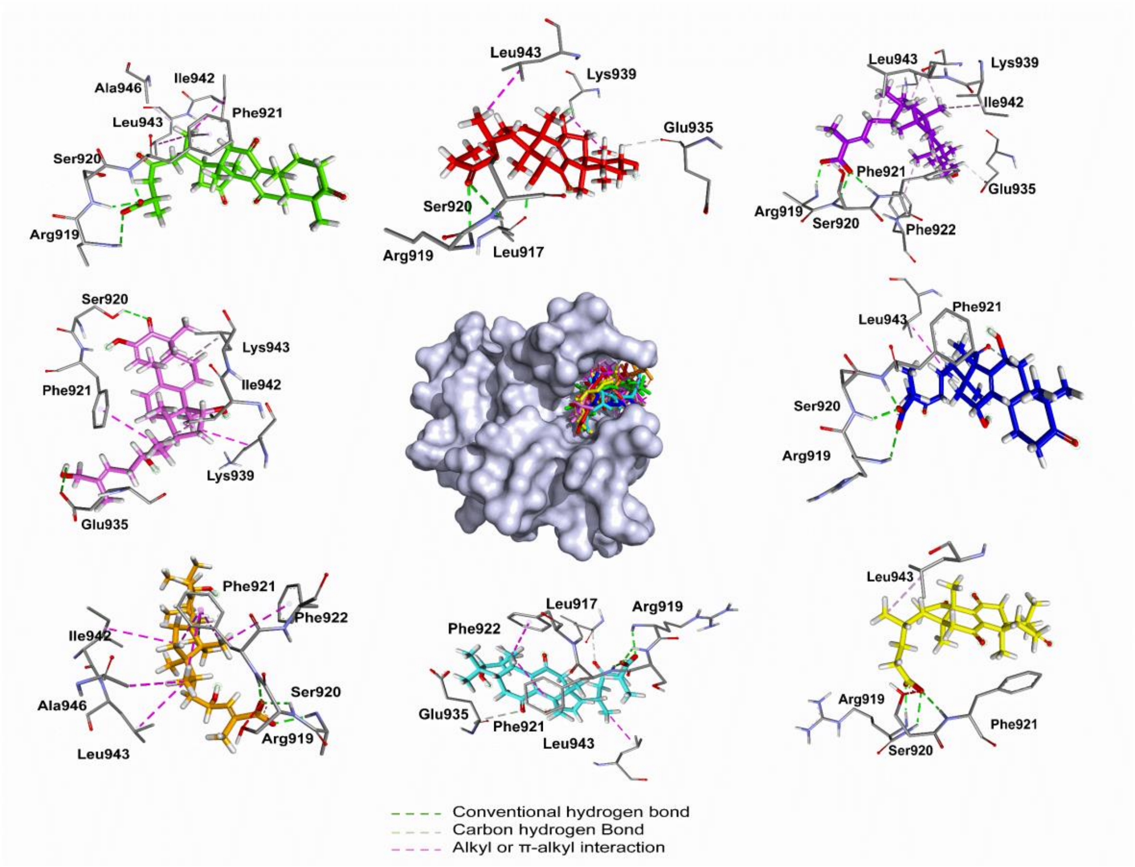

Figure S3. Result for the best molecular docking poses for the complexes MYC-**I** (green), MYC-**II** (red), MYC-**III** (violet), MYC-I**V** (blue), MYC-**V** (yellow), MYC-**VI** (cyan), MYC-**VII** (orange), and MYC-**VIII** (magent). Hydrogen bonds are represented in green, alkyl interactions in violet and salt bridges in orange.

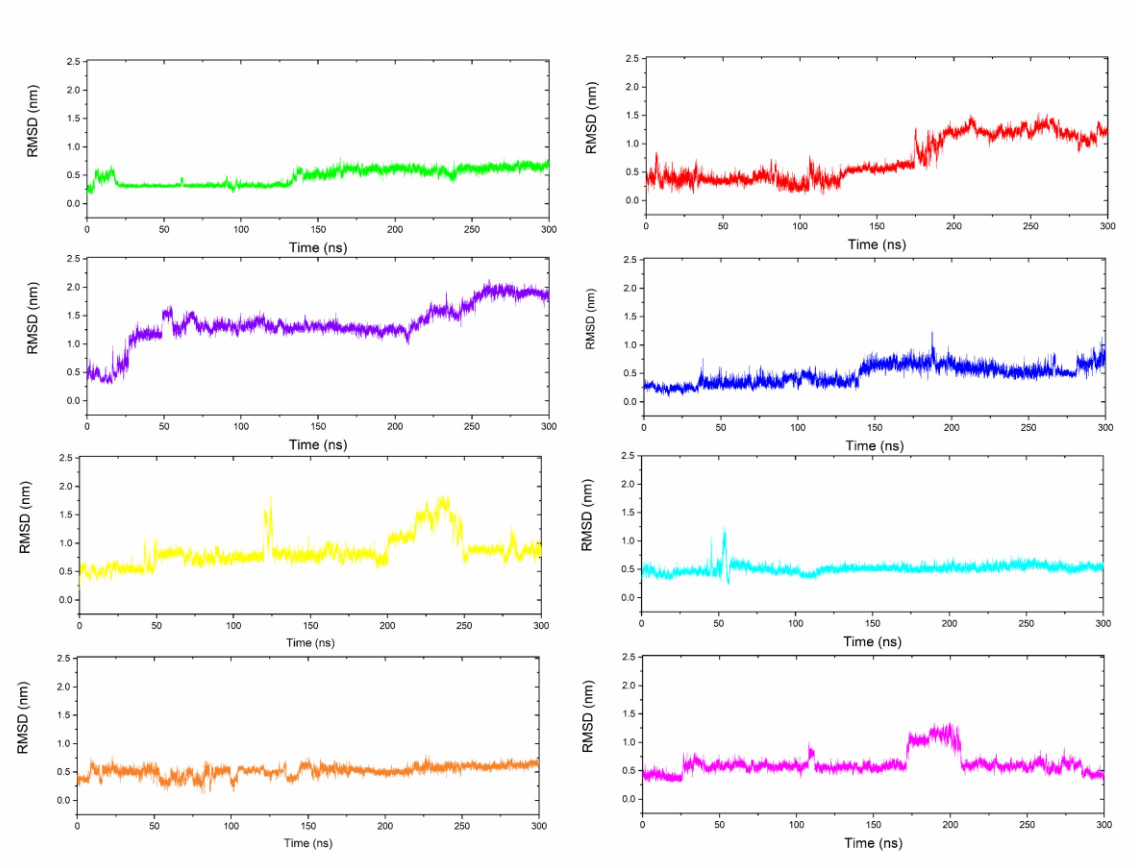

Figure S4. RMSD graph for the complexes MYC-**I** (green line), MYC-**II** (red line), MYC-**III** (violet line), MYC-**IV** (blue line), MYC-**V** (yellow line), MYC-**VI** (cyan line), MYC-**VII** (orange line), and MYC-**VIII** (magent line).

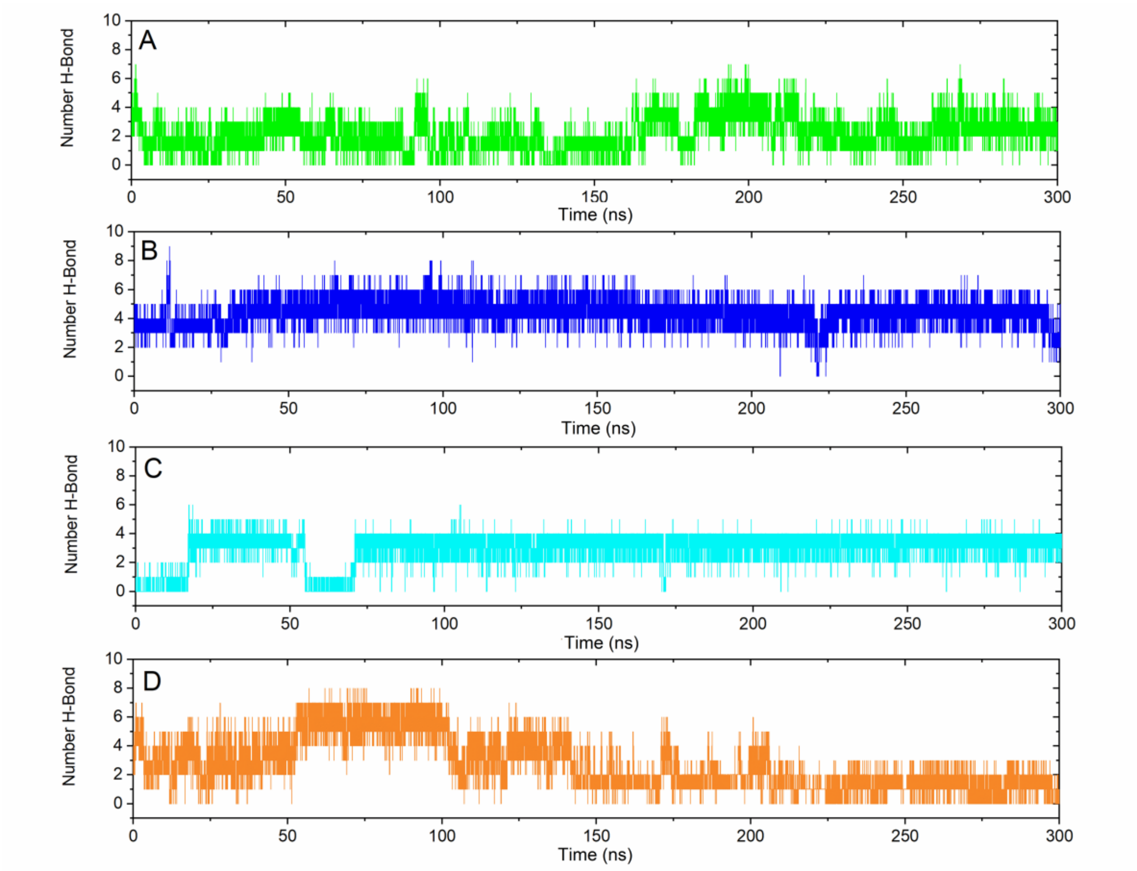

Figure S5. Hydrogen bond plots for the complex. (A) MYC-**I** (green line), (B) MYC-**IV** (blue line), (C) MYC-**VI** (cyan line), (D) MYC-**VII** (orange line)

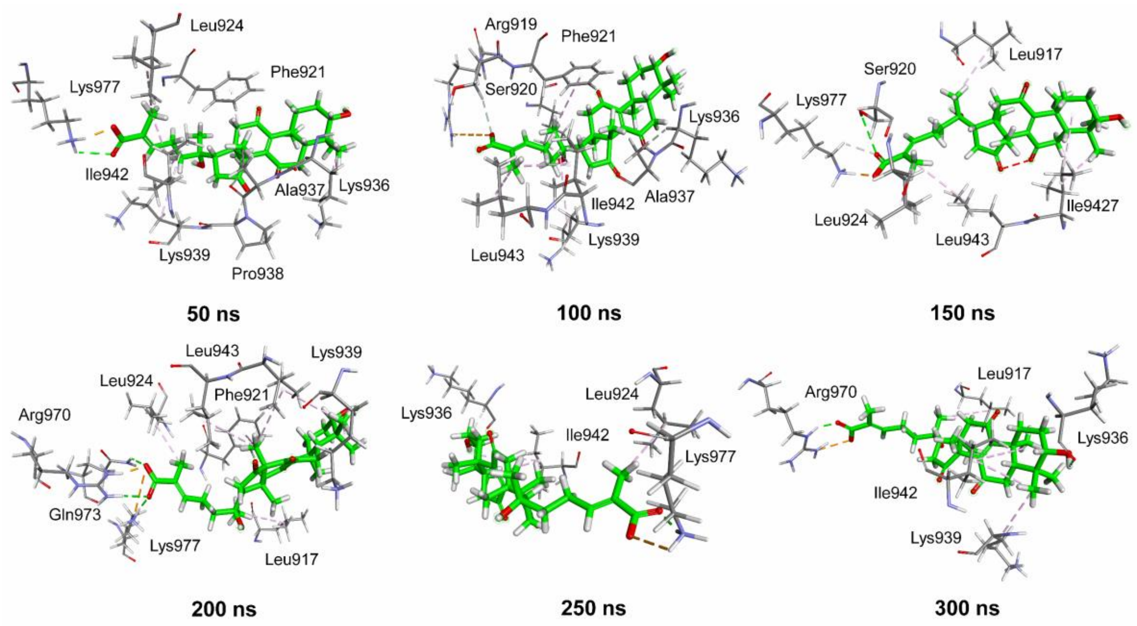

Figure S6. Interactions observed for the MYC-**I** complex during an MD trajectory at 50 ns, 100 ns, 150 ns, 200 ns, 250 ns and 300 ns. Hydrogen bonds are represented in green, alkyl interactions in violet, and salt bridges in orange.

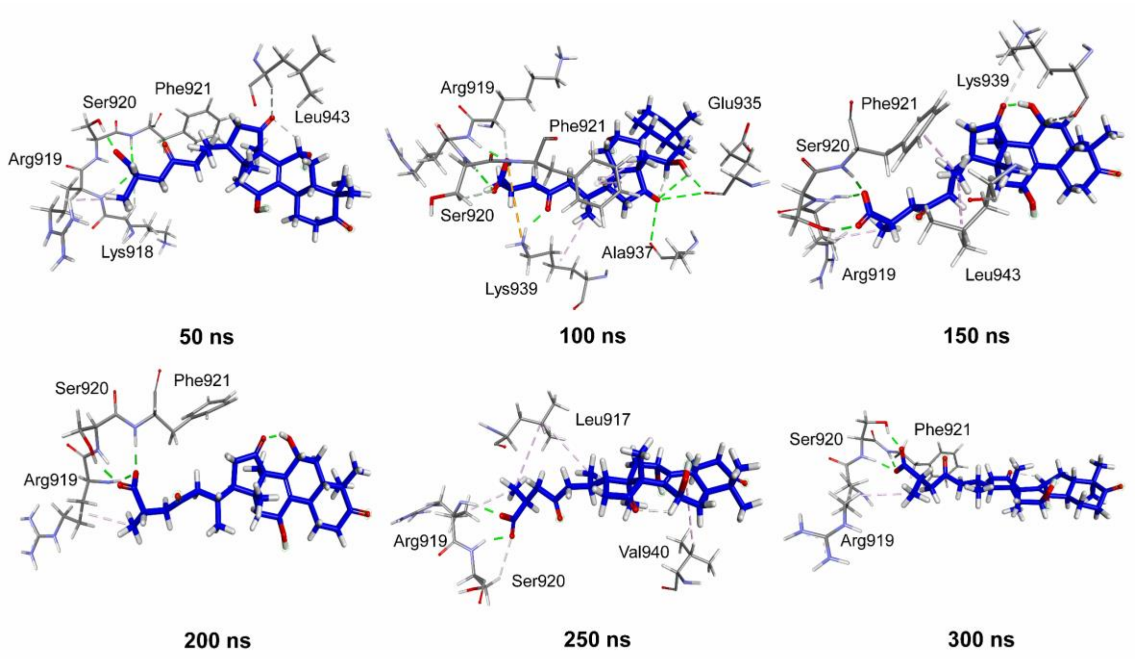

Figure S7. Interactions observed for the MYC-**IV** complex during an MD trajectory at 50 ns, 100 ns, 150 ns, 200 ns, 250 ns, and 300 ns. Hydrogen bonds are represented in green, alkyl interactions in violet and salt bridges in orange.

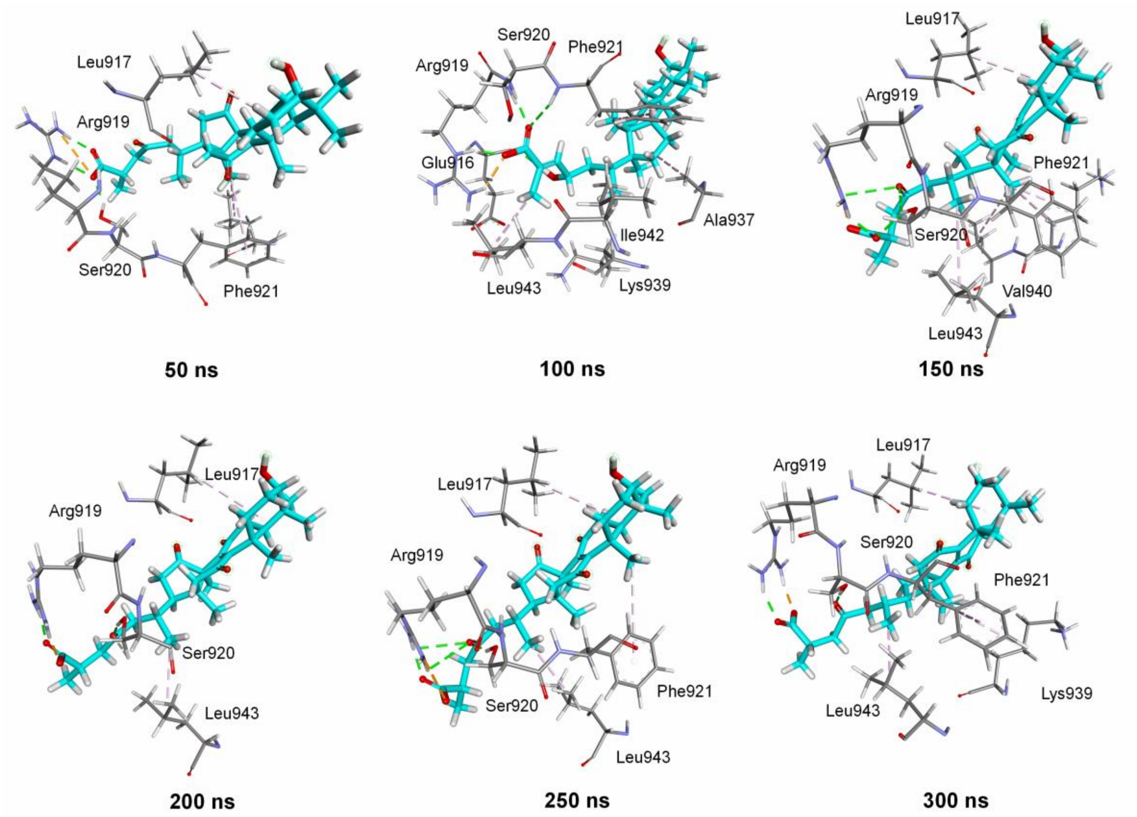

Figure S8. Interactions observed for the MYC-**VI** complex during an MD trajectory at 50 ns, 100 ns, 150 ns, 200 ns, 250 ns, and 300 ns. Hydrogen bonds are represented in green, alkyl interactions in violet, and salt bridges in orange.

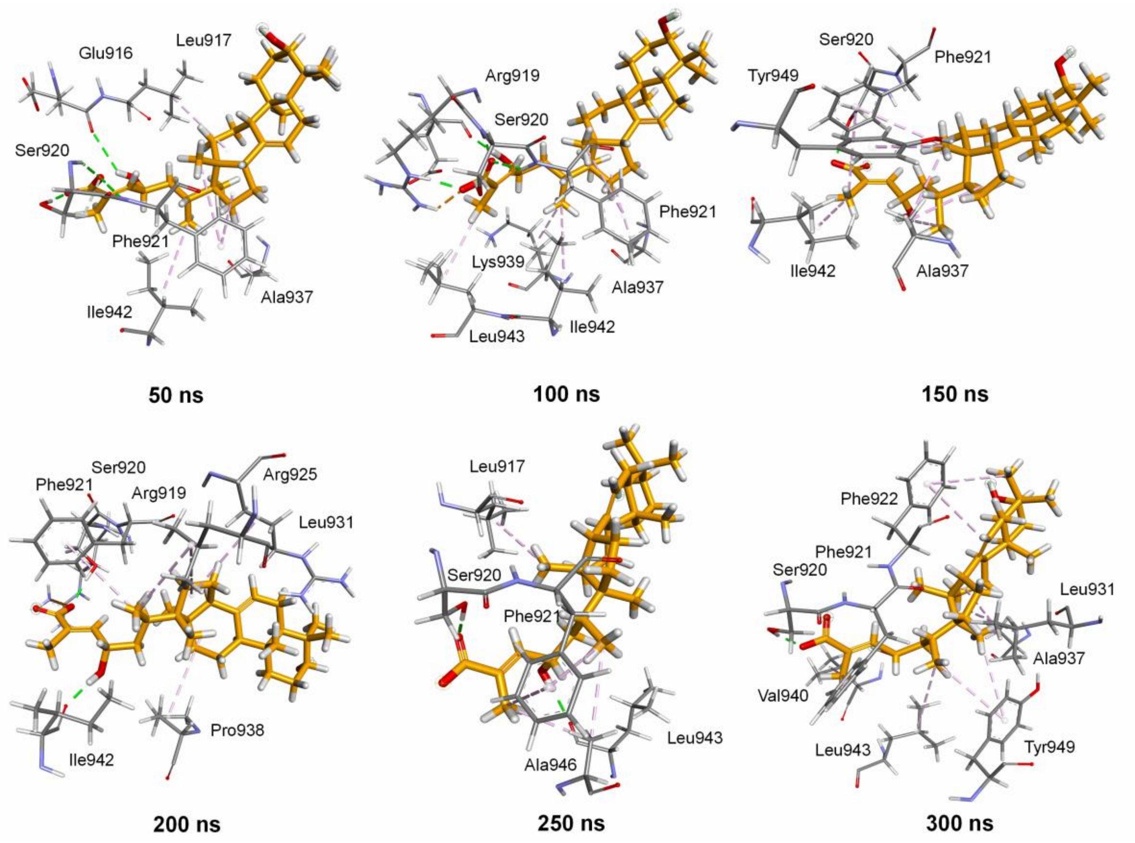

Figure S9. Interactions observed for the MYC-**VII** complex during an MD trajectory at 50 ns, 100 ns, 150 ns, 200 ns, 250 ns, and 300 ns. Hydrogen bonds are represented in green, alkyl interactions in violet and salt bridges in orange.

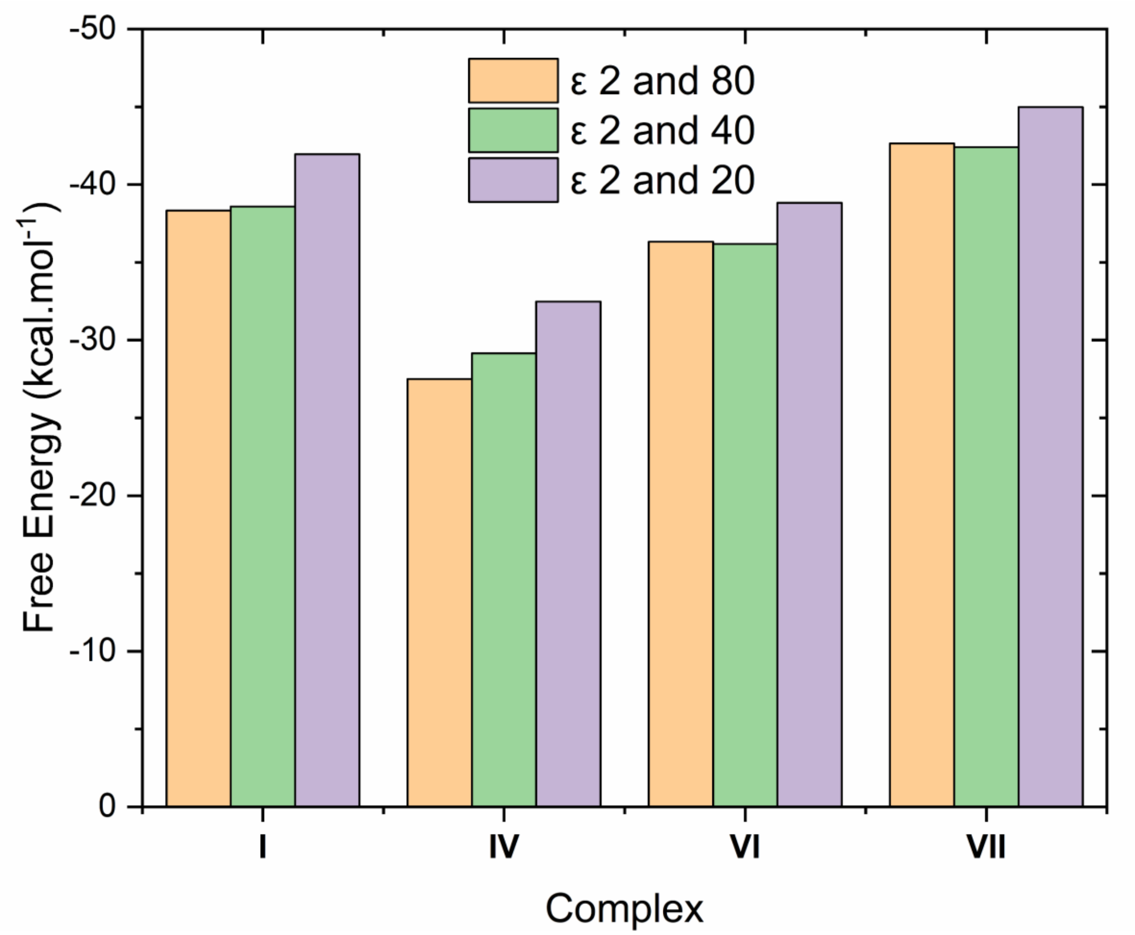

Figure S10. Influence of the external dielectric constant of the solvent on the calculation of free energy for values 20, 40, and 80.

Table S1. Binding site identified by the cavityplus server for clusters 0-4.

Cluster	DrugScore	Druggabilitty	Residues
0	1092.00	Strong	Gly982, Arg919, Leu943, Lys936, Gly983, Ala946, Gln912, Ile942, Lys918, Thr947, Arg925, Leu931, Val940, Leu924, Ser920, Cys984, Ala937, Phe921, Val941, Lys939, Lys944, Leu917, Glu916, Pro938, Phe922, Asn915, Asn934, Gln980, Glu935
1	175.00	Medium	Val908, Leu943, Lys936, Leu909, Gln912, Ile942, Lys918, Arg925, Leu931, Val940, Arg904, Ala937, Thr905, Phe921, Val941, Lys939, Leu917, Glu916, Pro938, Phe922, Asn915, Asn934, Val901, Glu935
2	-699.00	Weak	Leu931, Val940, Lys918, Pro938, Phe922, Asn933, Leu943, Asn915, Glu932, Asn934, Ala937, Ile942, Phe921, Lys939, Glu935, Val941, Arg925
3	-560,00	Weak	Leu966, Ile928, Arg919, Ile961, Glu932, Glu910, His906, Ser952, Asp965, Arg903, Leu909, Gln958, Glu964, Gln973, His976, Ser962, Pro929, Arg925, Lys902, Arg971, Glu972, Asn907, Val953, Met899, Leu924, Ser920, Gln954, Arg904, Lys969, Leu978, Arg913, Cys984, Thr905, Lys975, Glu979, Asn900, Ala923, asp926, Leu974, Gln927, Phe922, Glu930, Arg968, Lys977, Val901, Gln980
4	-780.00	Weak	His898, Gly897, Ile928, Val908, Arg919, Glu932, Glu910, His906, Arg903, Leu909, Gln973, His976, Glu972, Arg925, Lys902, Arg971, Leu931, Asn907, Met899, Leu924, Arg904, Lys969, Arg913, Thr905, Phe921, Lys975, Ala923, Asp926, Leu974, Gln927, Phe922, Asn933, Arg968, Asn934, Val901

Table S2. Interaction energy results for the 82 ligands selected by induced coupling and inhibitors described in the literature.

Ligand	Energy (kcal.mol^{-1})	Cluster	Chemical structure	Reference
9-ene-C16	-10.1	1	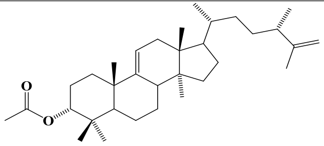	1
8-ene-epoxy-new-C2	-9.4	0	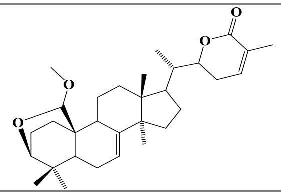	2
7(8)-ene-C16	-9.1	0	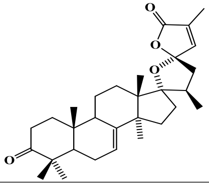	3
7(8)-ene-C15	-9.0	0	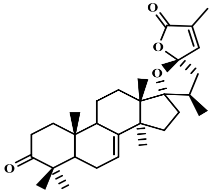	3

7(8)-ene-C11	-8.9	0	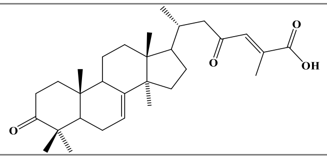	3
7(8)-ene-C3	-8.9	0	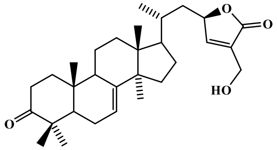	4
8-ene-C60	-8.9	0	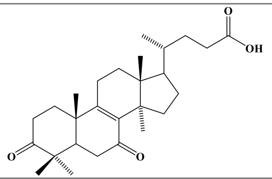	5
8-ene-C179	-8.8	0	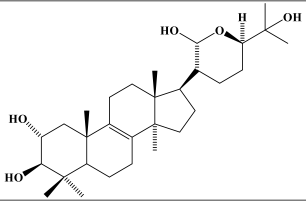	6
7(8)-ene-C27	-8.7	0	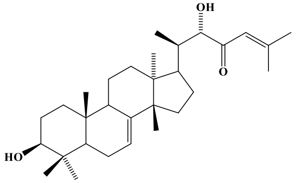	7
7(8)-ene-C4	-8.7	0	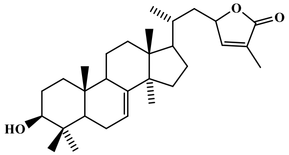	8

7,9-diene-C120	-8.7	0	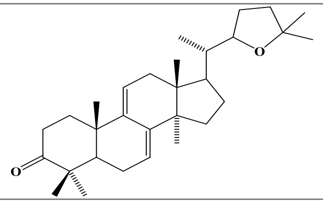	9
8-ene-C137	-8.7	0	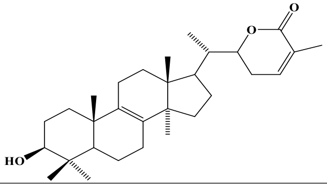	2
8-ene-C157	-8.7	0	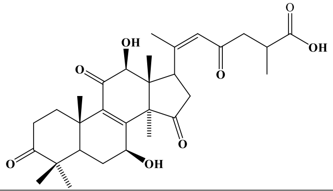	10
8-ene-C186	-8.7	0	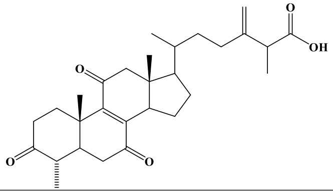	11
8-ene-C353	-8.7	0	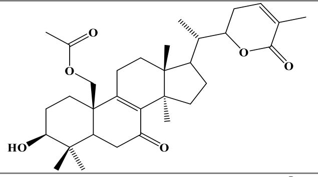	12
7,9-diene-C22	-8.6	0	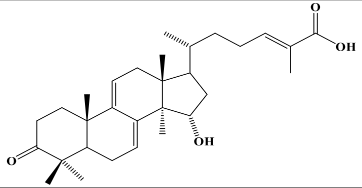	13

8,11(12)-diene-C2	-8.6	0	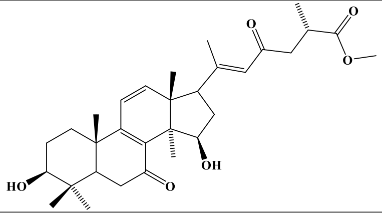	14
8-ene-C188	-8.6	0	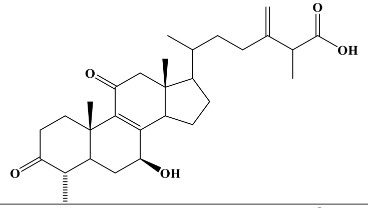	11
8-ene-C287	-8.6	0	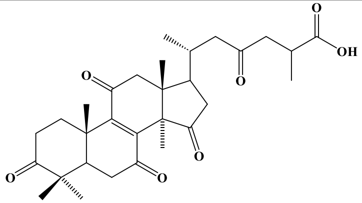	15
8-ene-C344	-8.6	0	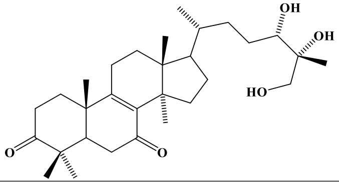	16
8-ene-C434	-8.6	0	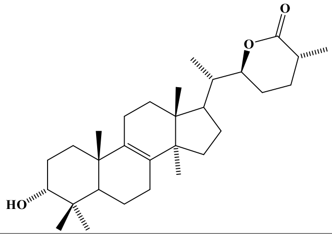	17

8-ene-C47	-8.6	0	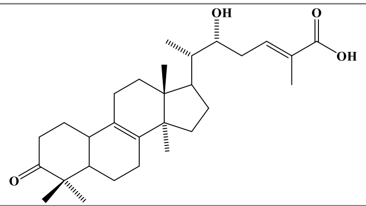	17
7(8)-ene-C19	-8.5	0	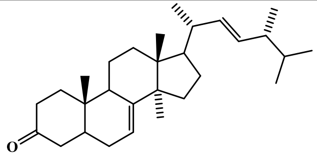	18
7,14-diene-exo-C3	-8.5	0	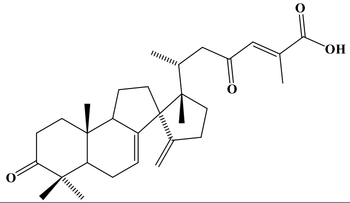	3
7,9-diene-C125	-8.5	0	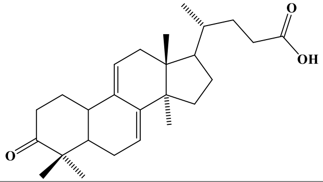	19
7,9-diene-C91	-8.5	0	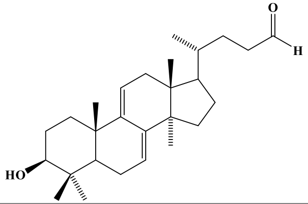	20
7,9-diene-lactone-C1	-8.5	0	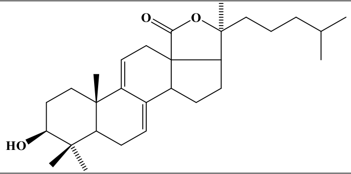	21

8-ene-C114	-8.5	0	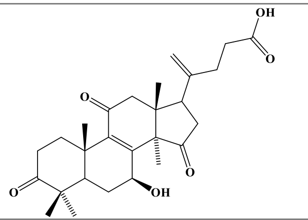	22
8-ene-C135	-8.5	0	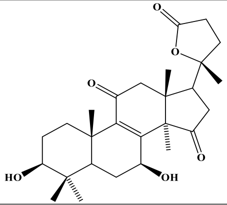	23
8-ene-C138	-8.5	0	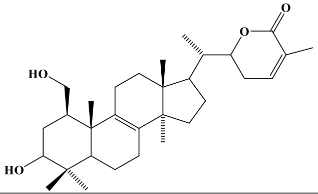	2
8-ene-C163	-8.5	0	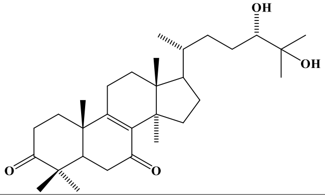	24
8-ene-C284	-8.5	0	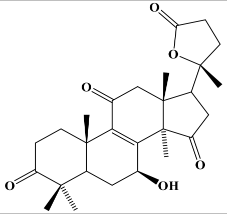	23

8-ene-C314	-8.5	0	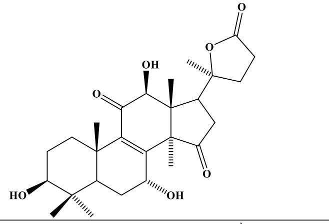	25
8-ene-C382	-8.5	0	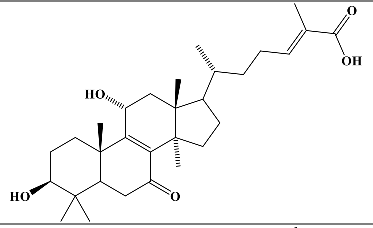	26
8-ene-new-C1	-8.5	0	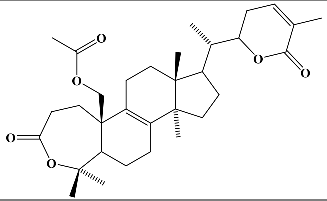	27
9,16-diene-epoxy-C6	-8.5	0	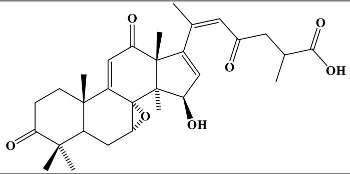	28
9-ene-epoxy-C4	-8.5	0	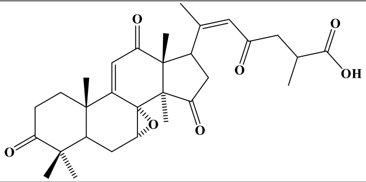	29

7,9-diene-C113	-8.4	0	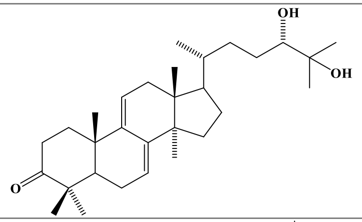	30
7,9-diene-C14	-8.4	0	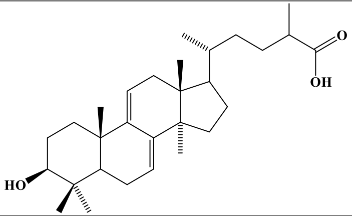	31
7,9-diene-C20	-8.4	0	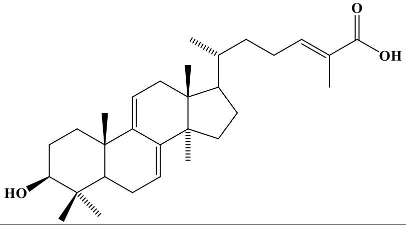	32
8-ene-C107	-8.4	0	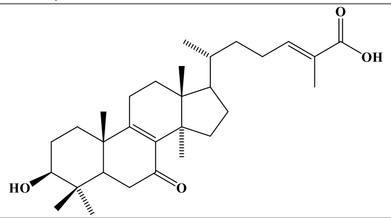	33
8-ene-C219	-8.4	0	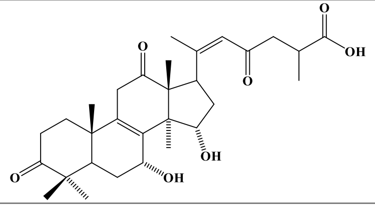	34

8-ene-C295	-8.4	0	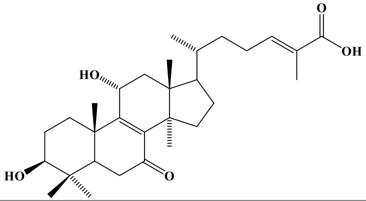	26
8-ene-C300	-8.4	0	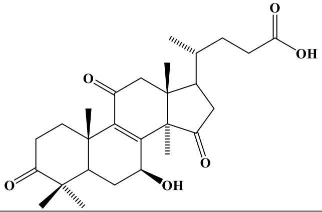	35
8-ene-C342	-8.4	0	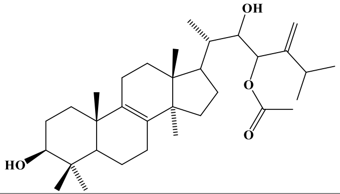	36
8-ene-C368	-8.4	0	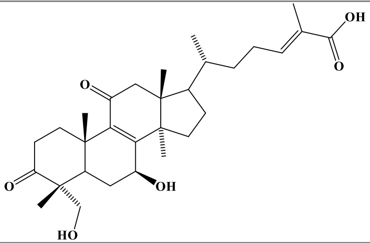	37
8-ene-C375	-8.4	0	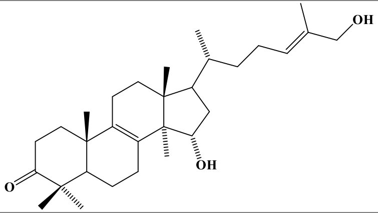	38

8-ene-C68	-8.4	0	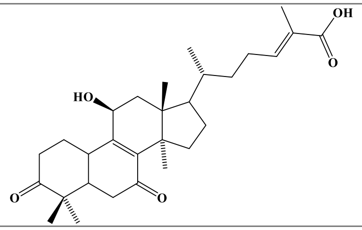	5
saturated-C6	-8.4	0	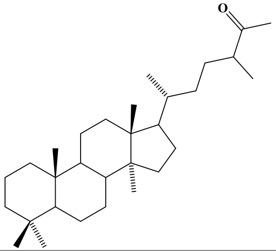	39
5,7-diene-demethylated-C1	-8.3	0	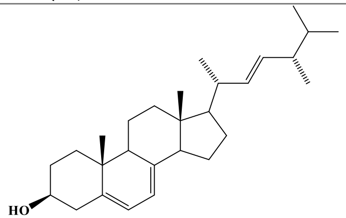	17
6-ene-peroxy-C1	-8.3	0	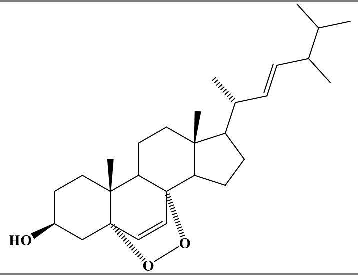	40

7(8)-ene-C18	-8.3	0	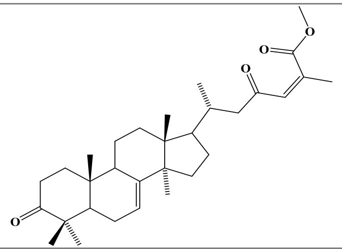	3
7(8)-ene-C6	-8.3	0	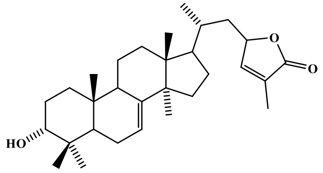	8
7(8)-ene-C7	-8.3	0	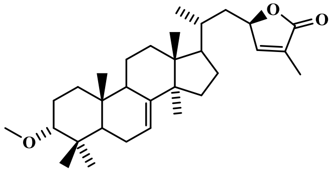	8
7,12-diene-C4	-8.3	0	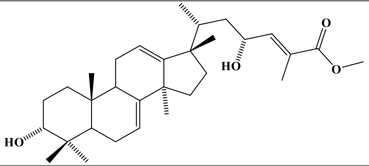	3
7,14-diene-C1	-8.3	0	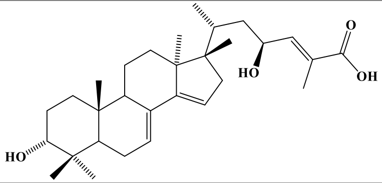	3

7,9-diene-C132	-8.3	0	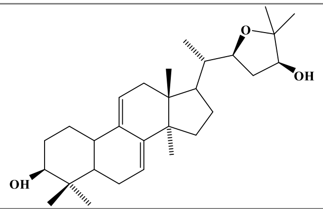	41
7,9-diene-C133	-8.3	0	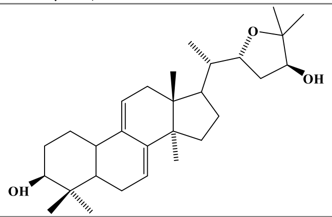	41
1,7,9-triene-C4	-8.3	0	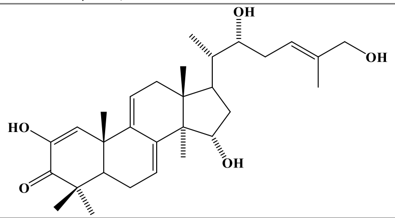	42
7,9-diene-C43	-8.3	0	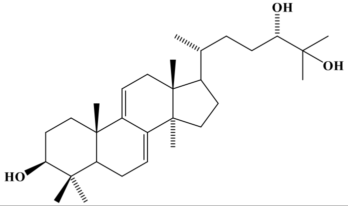	43
7,9-diene-C57	-8.3	0	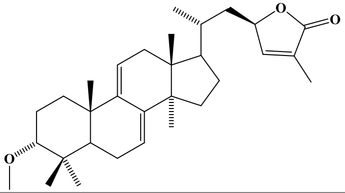	8

7,9-diene-C6	-8.3	0	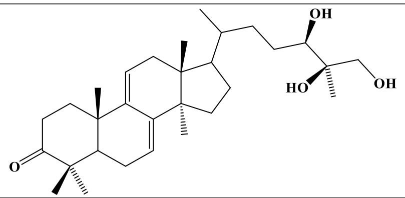	44
7,9-diene-C76	-8.3	0	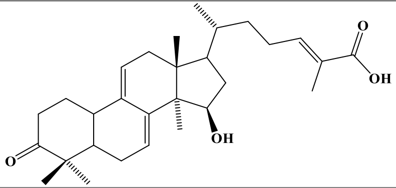	45
7,9-diene-C92	-8.3	0	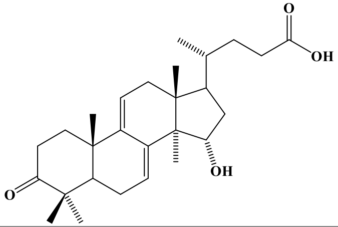	31
7,9-diene-C96	-8.3	0	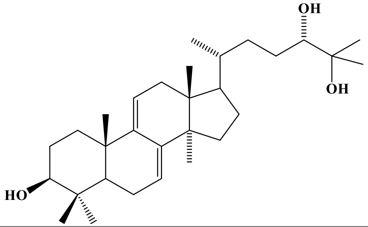	30
7,9-diene-demethylated-C3	-8.3	0	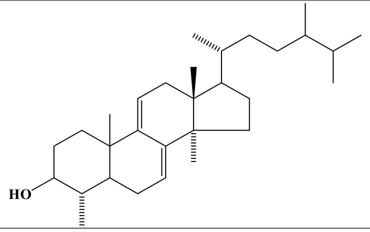	46

7-ene-demethylated-C4	-8.3	0	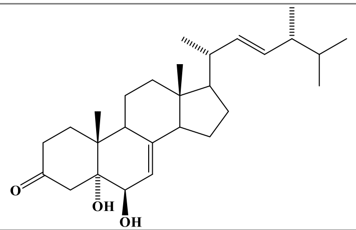	47
8,16-diene-C6	-8.3	0	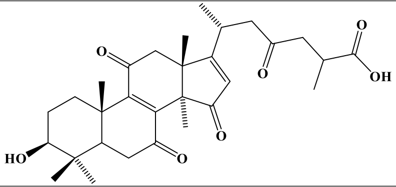	26
8-ene-7,15-epoxy-C2	-8.3	0	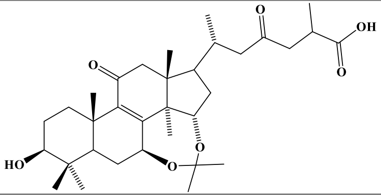	48
8-ene-C104	-8.3	0	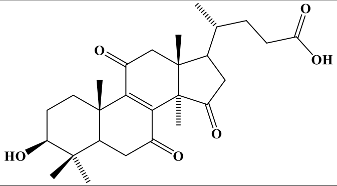	49
8-ene-C187	-8.3	0	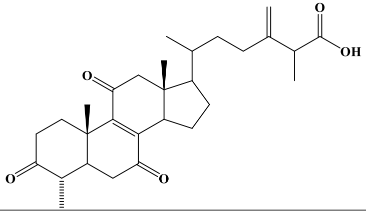	11

8-ene-C223	-8.3	0	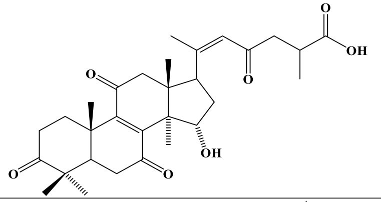	50
8-ene-C325	-8.3	0	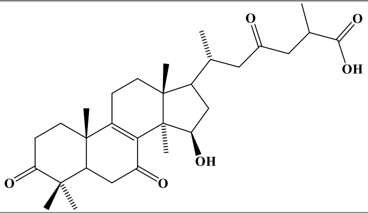	16
8-ene-C369	-8.3	0	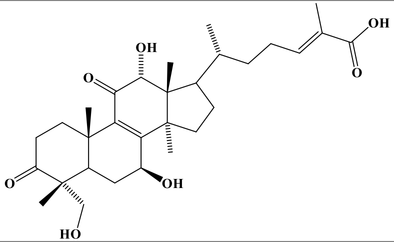	37
8-ene-C370	-8.3	0	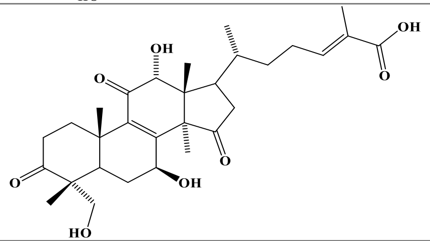	37
8-ene-C420	-8.3	0	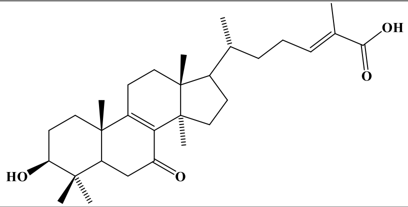	33

8-ene-C421	-8.3	0	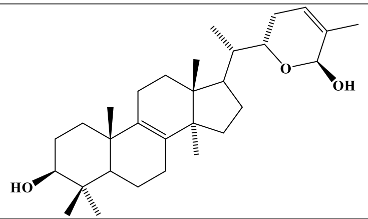	51
8-ene-C439	-8.3	0	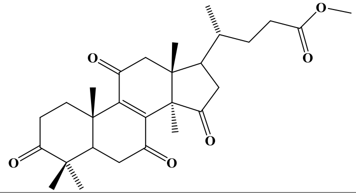	15
8-ene-C58	-8.3	0	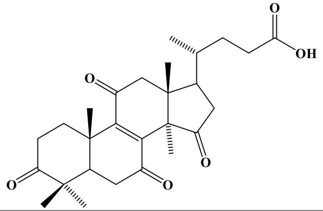	15
8-ene-C69	-8.3	0	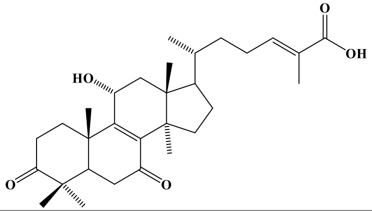	5
8-ene-C72	-8.3	0	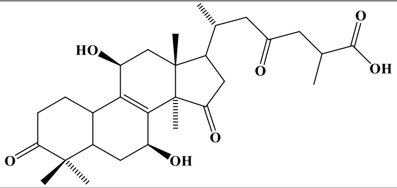	52

9,16-diene-epoxy-C15	-8.3	0	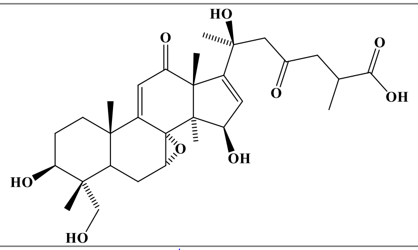	53
D347-2761	-7.6	-	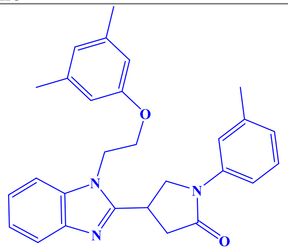	54
7594-0037	-6.8	-	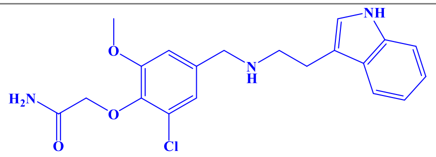	55
L755507	-5.6	-	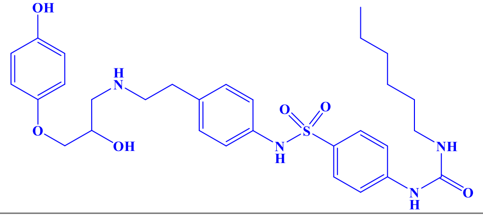	56
10074-65	-8.3	-	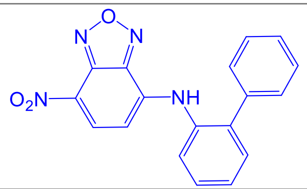	57

Table S3. Druglikeness and physiochemical properties for the 82 selected ligands.

Ligand	MF	MW	cLog P	HBA	HBD	TPSA	RB	Log S	Log $D_{7.4}$	Lipinski	Veber
7(8)-ene-C11	$C_{30}H_{44}O_4$	468.678	6.787	4	1	71.44	5	-5.91	2.01	Yes	Yes
7(8)-ene-C15	$C_{30}H_{42}O_4$	466.662	6.539	4	0	52.60	0	-6.17	4.08	Yes	Yes
7(8)-ene-C16	$C_{30}H_{42}O_4$	466.662	6.539	4	0	52.60	0	-6.17	4.08	Yes	Yes
7(8)-ene-C19	$C_{29}H_{46}O$	410.686	8.009	1	0	17.07	4	-6.98	5.02	Yes	Yes
7(8)-ene-C27	$C_{30}H_{48}O_3$	456.711	6.485	3	2	57.53	4	-6.19	4.36	Yes	Yes
7(8)-ene-C3	$C_{30}H_{44}O_4$	468.678	6.031	4	1	63.60	4	-6.04	3.92	Yes	Yes
7(8)-ene-C4	$C_{30}H_{46}O_3$	454.695	6.852	3	1	46,53	3	-6.25	4.16	Yes	Yes
7(8)-ene-C6	$C_{30}H_{46}O_3$	454.695	6.852	3	1	46,53	3	-6.25	4.16	Yes	Yes
7,12-diene-C4	$C_{31}H_{48}O_4$	484.721	6.379	4	2	66.76	6	-5.84	3.76	Yes	Yes
7,14-diene-exo-C3	$C_{30}H_{42}O_4$	466.662	6.707	4	1	71.44	3	-5.85	1.99	Yes	Yes
7,9-diene-C113	$C_{30}H_{48}O_3$	456.711	6.629	3	2	57.53	5	-6.17	4.53	Yes	Yes
7,9-diene-C120	$C_{30}H_{46}O_2$	438696	7.675	2	0	26.30	2	-6.33	5.04	Yes	Yes
7,9-diene-C125	$C_{26}H_{38}O_3$	398.587	6.192	3	1	54.37	4	-6.03	2.04	Yes	Yes
7,9-diene-C132	$C_{29}H_{46}O_3$	442.684	6.047	3	2	49.69	2	-6.44	4.30	Yes	Yes
7,9-diene-C133	$C_{29}H_{46}O_3$	442.684	6.047	3	2	49.69	2	-6.44	4.30	Yes	Yes
7,9-diene-C20	$C_{30}H_{46}O_3$	454.695	7.320	3	2	57.53	5	-6.16	2.09	Yes	Yes
7,9-diene-C43	$C_{30}H_{50}O_3$	458.727	6421	3	3	60.69	5	-6.18	4.34	Yes	Yes
7,9-diene-C76	$C_{29}H_{42}O_4$	454.651	6.109	4	2	74.60	5	-5.95	1.97	Yes	Yes
7,9-diene-C91	$C_{27}H_{42}O_2$	398.631	6.488	3	1	37.30	4	-5.99	4.55	Yes	Yes
7,9-diene-C92	$C_{27}H_{40}O_4$	428.613	5.553	4	2	74.60	4	-6.04	2.02	Yes	Yes
7,9-diene-C96	$C_{30}H_{50}O_3$	458.727	6.421	3	3	60.69	4	-6.18	4.34	Yes	Yes
7,9-diene-demethylated-C3	$C_{30}H_{50}O$	426.729	8.191	1	1	20.23	5	-6.61	4.64	Yes	Yes
7,9-diene-lactone-C1	$C_{29}H_{44}O_3$	440.668	6.604	3	1	46.53	4	-6.19	4.21	Yes	Yes
8-ene-C107	$C_{30}H_{46}O_4$	470.694	6.723	4	2	74.60	5	-5.91	2.00	Yes	Yes
8-ene-C135	$C_{27}H_{38}O_6$	458.595	3.521	6	2	100.90	1	-4.95	3.30	Yes	Yes
8-ene-C137	$C_{30}H_{46}O_3$	454.695	6.995	3	1	46.53	2	-6.38	4.19	Yes	Yes
8-ene-C138	$C_{31}H_{48}O_4$	484.721	6.213	4	2	66.76	3	-5.82	3.92	Yes	Yes
8-ene-C163	$C_{30}H_{48}O_4$	472.710	6.032	4	2	74.60	5	-5.89	4.23	Yes	Yes
8-ene-C284	$C_{27}H_{36}O_6$	456.579	3.729	6	1	97.74	1	-4.95	3.26	Yes	Yes

8-ene-C314	$C_{27}H_{38}O_7$	474.594	2.482	7	3	121.13	1	-4.43	2.35	Yes	Yes
8-ene-C344	$C_{30}H_{48}O_5$	488.709	5.004	5	3	94.83	6	-5.61	4.03	Yes	Yes
8-ene-C420	$C_{30}H_{46}O_4$	470.694	6.723	4	2	74.60	5	-5.91	2.07	Yes	Yes
8-ene-C421	$C_{30}H_{48}O_3$	456.711	6.786	3	2	49.69	2	-6.44	4.47	Yes	Yes
8-ene-C434	$C_{30}H_{48}O_3$	456.711	7.074	3	1	46.53	2	-6.37	4.47	Yes	Yes
8-ene-C47	$C_{29}H_{44}O_4$	456.667	6.333	4	2	74.60	5	-6.03	1.96	Yes	Yes
8-ene-C69	$C_{30}H_{44}O_5$	484.677	5.902	5	2	91.67	5	-5.68	1.95	Yes	Yes
5,7-diene-demethylated-C1	$C_{28}H_{44}O$	396.659	7.331	1	1	20.23	4	-654	4.12	Yes	Yes
6-ene-peroxy-C1	$C_{28}H_{44}O_3$	442.684	6.864	3	1	36.69	4	-6.54	4.57	Yes	Yes
7(8)-ene-C18	$C_{31}H_{46}O_4$	482.705	6.875	4	0	60.44	6	-6.98	5.02	Yes	Yes
7(8)-ene-C7	$C_{31}H_{48}O_3$	468.722	7.505	3	0	35.53	3	-6.35	4.52	Yes	Yes
7,14-diene-C1	$C_{30}H_{46}O_4$	470.694	6.291	4	3	77.76	5	-5.88	2.04	Yes	Yes
7,9-diene-C14	$C_{29}H_{46}O_3$	442.684	7.010	3	2	57.53	5	-6.09	2.12	Yes	Yes
7,9-diene-C22	$C_{30}H_{44}O_4$	468.678	6.499	4	2	74.60	5	-5.80	1.98	Yes	Yes
1,7,9-triene-C4	$C_{30}H_{44}O_5$	484.677	5.039	5	4	97.99	5	-5.56	2.11	Yes	Yes
7,9-diene-C57	$C_{31}H_{46}O_3$	466.706	7.425	3	0	35.53	4	-6.30	4.32	Yes	Yes
7,9-diene-C6	$C_{30}H_{48}O_4$	472.710	5.601	4	3	77.76	6	-5.88	4.29	Yes	Yes
7-ene-demethylated-C4	$C_{28}H_{44}O$	396.659	7.331	1	1	20.23	4	-6.29	4.39	Yes	Yes
8,11(12)-diene-C2	$C_{31}H_{44}O_6$	512.687	4.737	6	2	100.90	6	-5.19	2.79	Yes	Yes
8,16-diene-C6	$C_{32}H_{44}O_9$	512.643	4.260	7	2	125.81	6	-5.05	1.44	Yes	Yes
8-ene-7,15-epoxy-C2	$C_{33}H_{50}O_7$	558.756	5.722	7	2	110.13	6	-5.46	2.39	No	Yes
8-ene-C104	$C_{27}H_{38}O_6$	458.595	4.135	6	2	108.74	4	-5.13	1.44	Yes	Yes
8-ene-C114	$C_{27}H36O_6$	456.579	4.055	6	2	108.74	4	-5.09	1.42	Yes	Yes
8-ene-C157	$C_{30}H_{40}O_8$	528.642	3.231	8	3	146.04	5	-4.33	0.99	Yes	No
8-ene-C179	$C_{30}H_{50}O_5$	490.725	4.952	5	4	90.15	2	-5.65	3.94	Yes	Yes
8-ene-C186	$C_{29}H_{42}O_4$	454.651	6.397	4	1	71.44	6	-5.90	1.95	Yes	Yes
8-ene-C187	$C_{29}H_{40}O_5$	468.634	5.576	5	1	88.51	6	-5.91	1.84	Yes	Yes
8-ene-C188	$C_{29}H_{42}O_5$	470.650	5.368	5	2	91.67	6	-5.88	1.86	Yes	Yes
8-ene-C219	$C_{30}H_{42}O_7$	514.659	4.052	7	3	128.97	5	-4.98	1.52	Yes	Yes
8-ene-C223	$C_{30}H_{40}O_7$	512.643	4.260	7	2	125.81	5	-5.07	1.59	Yes	Yes
8-ene-C287	$C_{30}H_{40}O_7$	512.643	4.548	7	1	122.65	6	-5.20	1.30	Yes	Yes
8-ene-C295	$C_{30}H_{46}O_5$	486.693	5.694	5	3	94.83	5	-5.75	2.03	Yes	Yes
8-ene-C300	$C_{27}H_{38}O_6$	458.595	4.135	6	2	108.74	4	-5.09	1.52	Yes	Yes
8-ene-C325	$C_{30}H_{44}O_6$	500.676	5.161	6	2	108.74	5	-5.59	1.93	No	Yes
8-ene-C342	$C_{33}H_{54}O_4$	514.791	7.238	4	2	66.76	6	-5.83	4.25	No	Yes

8-ene-C353	$C_{32}H_{46}O_6$	526.714	5.717	6	1	89.90	5	-5.77	3.92	No	Yes
8-ene-C368	$C_{30}H_{44}O_6$	500.676	4.874	6	3	111.90	6	-5.41	1.81	Yes	Yes
8-ene-C369	$C_{30}H_{44}O_7$	516.675	3.845	7	4	132.13	6	-4.89	1.35	Yes	Yes
8-ene-C370	$C_{30}H_{42}O_8$	530.658	3.024	8	4	149.20	6	-4.10	0.63	Yes	No
8-ene-C375	$C_{30}H_{48}O_3$	456.711	6.631	3	2	57.53	5	-6.20	4.48	Yes	Yes
8-ene-C382	$C_{30}H_{46}O_5$	486.693	5.694	5	3	94.83	5	-5.75	2.03	Yes	Yes
8-ene-C439	$C_{28}H_{38}O_6$	470.606	4.431	6	0	94.58	5	-5.21	2.46	No	Yes
8-ene-C58	$C_{27}H_{36}O_6$	456.579	4.343	6	1	105.58	4	-5.11	1.42	Yes	Yes
8-ene-C60	$C_{27}H_{40}O_4$	428.613	5.985	4	1	71.44	4	-6.04	2.00	Yes	Yes
8-ene-C68	$C_{24}H_{49}O_5$	470.650	5.512	5	2	91.67	5	-5.84	1.86	Yes	Yes
8-ene-C72	$C_{29}H_{42}O_7$	502.648	3.741	7	3	128.97	6	-4.97	1.37	Yes	Yes
8-ene-epoxy-new-C2	$C_{31}H_{46}O_4$	482.705	6.841	4	0	44.76	3	-6.15	4.30	Yes	Yes
8-ene-new-C1	$C_{32}H_{46}O_6$	526.714	6.472	6	0	78.90	5	-5.56	3.83	No	Yes
9,16-diene-epoxy-C6	$C_{30}H_{38}O_7$	510.627	3.988	7	2	121.27	5	-4.88	1.64	Yes	Yes
9-ene-epoxy-C4	$C_{30}H_{38}O_7$	510.627	4.276	7	1	118.11	5	-5.08	1.74	Yes	Yes
saturated-C6	$C_{30}H_{52}O$	428.745	8.703	1	0	17.07	5	-7.05	5.21	Yes	Yes
9-ene-C16	$C_{33}H_{54}O_2$	482.793	9.152	2	0	26.30	7	-6.47	4.68	Yes	Yes
9,16-diene-epoxy-C15	$C_{30}H_{42}O_9$	546.657	1.947	9	5	164.89	6	-3.68	0.57	Yes	No

Molecular formula (MF), partition coefficient (cLog P), molecular weight (MW, Daltons), hydrogen bond acceptors (HBA), hydrogen bond donors (HBD), topological polar surface area (TPSA, Å^2), number of rotatable bonds (RB), solubility (Log S, log mol.L^{-1}), and distribution coefficient (Log $D_{7.4}$).

Table S4. Pharmacokinetic parameters for the 82 ligands selected by induced coupling.

Ligand	Pgpi (%)	Pgps (%)	GI-A (%)	F30 (%)	BBB (%)	T1/2 (h)	CL (h)	hERG (%)	H-HT (%)
7(8)-ene-C11	76.30	6.10	76.70	36.30	80.10	1.86	1.17	40.80	57.00
7(8)-ene-C15	81.20	6.20	73.80	39.20	88.60	2.00	1.40	55.10	49.40
7(8)-ene-C16	81.20	6.20	73.80	39.20	88.60	2.00	1.40	55.10	49.40
7(8)-ene-C19	92.30	10.40	89.30	43.50	97.90	2.00	0.93	47.30	7.00
7(8)-ene-C27	54.00	11.10	83.30	41.50	75.80	1.79	1.22	55.20	41.80
7(8)-ene-C3	79.90	12.20	71.30	32.60	87.70	1.99	1.38	48.8	57.20
7(8)-ene-C4	80.60	16.40	81.70	39.20	85.50	1.91	1.16	50.7	45.00
7(8)-ene-C6	80.60	16.40	81.70	39.20	85.50	1.91	1.16	50.7	45.00
7,12-diene-C4	89.90	8.70	70.50	32.60	35.20	1.92	1.29	41.90	46.60
7,14-diene-exo-C3	77.40	1.60	76.30	38.00	76.00	2.03	1.17	44.40	54.80
7,9-diene-C113	75.80	7.60	77.80	42.80	84.40	1.95	1.12	41.70	43.80
7,9-diene-C120	70.30	8.60	89.00	42.40	94.30	2.01	1.03	47.50	29.00
7,9-diene-C125	63.90	12.70	82.20	47.90	87.80	1.98	1.13	45.80	62.20
7,9-diene-C132	60.90	39.30	80.00	47.90	95.20	2.01	1.17	47.40	49.80
7,9-diene-C133	60.90	39.30	80.00	47.90	95.20	2.01	1.17	47.40	49.80
7,9-diene-C20	84.10	8.70	80.8	40.90	65.90	1.96	1.16	44.20	48.40
7,9-diene-C43	61.7	23.10	77.90	47.80	76.60	1.90	1.12	42.00	32.00
7,9-diene-C76	85.20	15.70	72.10	41.30	64.60	1.94	1.42	44.30	68.60
7,9-diene-C91	84.30	8.00	93.50	48.80	85.90	1.99	1.29	47.70	59.20
7,9-diene-C92	52.30	12.80	71.60	35.20	88.80	2.02	1.59	37.60	70.80
7,9-diene-C96	61.70	23.10	77.90	47.80	76.60	1.90	1.12	42.00	32.00
7,9-diene-demethylated-C3	78.70	34.40	93.10	49.00	87.50	1.93	1.11	47.20	4.20
7,9-diene-lactone-C1	74.90	18.60	81.70	35.30	81.50	1.79	1.38	53.40	52.80
8-ene-C107	91.10	5.50	71.60	40.80	58.50	1.69	1.36	42.50	65.20
8-ene-C135	51.60	2.50	70.80	41.90	96.60	1.47	1.95	38.00	58.40
8-ene-C137	81.10	4.30	82.20	44.90	90.30	1.79	1.23	50.70	50.60
8-ene-C138	87.90	8.60	68.40	41.20	87.40	1.27	1.75	47.30	53.60
8-ene-C163	85.50	6.10	71.60	41.10	77.60	1.67	1.29	44.60	51.20
8-ene-C284	51.70	3.40	73.80	39.00	97.50	1.59	1.87	38.80	54.20
8-ene-C314	58.80	2.80	70.00	45.00	93.90	1.54	1.87	36.50	43.00
8-ene-C344	79.90	7.90	65.00	39.90	60.40	1.84	1.83	60.00	37.00
8-ene-C420	91.10	5.50	71.60	40.80	58.50	1.69	1.36	42.50	65.20
8-ene-C421	62.90	7.10	79.60	44.20	83.60	1.81	1.21	47.80	46.80
8-ene-C434	74.50	4.40	82.20	42.80	74.70	1.65	1.27	49.90	40.60
8-ene-C47	89.90	5.10	72.10	45.70	33.10	1.74	1.34	40.00	65.00
8-ene-C69	90.30	7.10	72.10	35.50	57.90	1.61	1.54	33.80	65.20
5,7-diene-demethylated-C1	90.10	11.20	92.10	46.30	92.90	1.98	1.05	53.10	11.00
6-ene-peroxy-C1	75.00	31.60	80.30	48.30	91.20	1.91	1.22	48.80	21.20
7(8)-ene-C18	92.30	10.04	89.30	43.50	97.90	2.03	0.93	47.30	7.00
7(8)-ene-C7	92.80	28.10	79.30	40.80	74.20	1.85	1.18	50.50	39.40
7,14-diene-C1	71.60	11.40	71.70	40.50	35.60	1.94	1.27	38.90	44.60
7,9-diene-C14	57.80	10.40	80.80	43.10	88.70	1.89	1.11	40.20	51.60
7,9-diene-C22	84.30	8.90	72.10	32.70	77.90	1.98	1.39	41.20	69.40
1,7,9-triene-C4	47.50	34.50	78.90	39.20	22.90	1.69	1.61	38.00	35.80
7,9-diene-C57	93.00	34.50	79.30	48.40	82.22	1.95	1.29	51.50	42.40
7,9-diene-C6	68.20	9.80	65.00	41.50	69.90	1.97	1.23	41.40	47.40
7-ene-demethylated-C4	93.10	5.80	76.00	47.80	82.50	2.07	1.07	45.90	41.40

8,11(12)-diene-C2	81.50	26.40	70.30	30.10	86.40	1.82	1.58	38.70	70.20
8,16-diene-C6	41.20	7.00	71.60	31.80	89.80	1.74	1.64	34.70	43.60
8-ene-7,15-epoxy-C2	44.80	9.40	67.40	28.70	82.10	1.61	1.72	39.90	58.00
8-ene-C104	29.50	4.40	71.60	36.40	76.10	1.63	1.83	32.90	54.60
8-ene-C114	45.50	6.80	70.90	37.70	89.30	1.65	1.88	31.70	56.60
8-ene-C157	72.00	15.80	72.20	37.70	85.60	1.86	1.59	35.50	55.20
8-ene-C179	69.60	3.30	62.30	40.70	81.70	1.74	1.31	48.00	45.20
8-ene-C186	88.30	1.50	75.50	40.30	81.20	1.71	1.26	49.70	57.40
8-ene-C187	85.00	2.40	75.50	42.30	85.00	1.59	1.33	40.90	56.80
8-ene-C188	83.50	4.60	71.50	41.50	78.70	1.52	1.49	36.80	62.00
8-ene-C219	71.50	10.10	71.60	34.60	80.60	1.73	1.72	32.90	67.00
8-ene-C223	78.70	12.30	71.60	34.50	93.20	1.75	1.66	35.10	50.20
8-ene-C287	27.00	3.70	76.30	34.60	95.30	1.72	1.45	37.80	17.40
8-ene-C295	85.40	7.50	71.60	39.40	58.30	1.68	1.59	35.60	72.40
8-ene-C300	51.90	8.10	71.30	36.80	88.60	1.63	1.86	32.90	55.60
8-ene-C325	58.40	8.50	71.60	31.00	88.40	1.66	1.63	35.20	55.60
8-ene-C342	81.90	1.70	72.50	38.10	39.80	1.70	1.20	45.60	42.20
8-ene-C353	89.40	7.10	67.20	31.80	75.10	1.60	1.57	41.30	55.60
8-ene-C368	84.70	8.30	74.00	34.70	53.90	1.64	1.68	34.50	64.80
8-ene-C369	88.40	10.00	74.80	35.70	41.20	1.59	1.73	33.50	65.60
8-ene-C370	87.90	12.00	74.80	34.60	54.60	1.73	1.64	34.10	51.60
8-ene-C375	91.40	4.10	85.00	39.60	52.50	1.83	1.23	55.20	45.40
8-ene-C382	85.40	7.50	71.60	39.40	58.30	1.68	1.59	35.60	72.40
8-ene-C439	75.00	3.50	77.60	31.40	95.70	1.71	1.47	38.30	33.20
8-ene-C58	40.90	4.00	74.70	33.30	93.30	1.67	1.57	36.90	29.80
8-ene-C60	78.60	6.50	74.70	39.00	83.30	1.68	1.40	43.30	64.60
8-ene-C68	88.10	11.70	72.10	48.80	55.30	1.60	1.54	37.00	81.20
8-ene-C72	38.70	17.50	71.60	46.00	86.00	1.62	1.75	34.50	46.00
8-ene-epoxy-new-C2	83.80	16.60	72.00	41.30	74.80	1.68	1.33	52.20	38.80
8-ene-new-C1	93.90	5.00	66.00	35.00	79.50	1.58	1.43	46.00	61.20
9,16-diene-epoxy-C6	76.20	23.60	68.30	34.20	77.20	1.92	1.72	38.20	66.20
9-ene-epoxy-C4	79.20	13.20	70.60	39.40	93.50	1.92	1.94	39.70	62.00
Saturated-C6	83.20	4.40	88.80	47.80	95.50	1.96	1.15	45.50	5.20
9-ene-C16	89.30	2.80	84.60	34.40	77.70	1.78	0.96	45.51	19.80
9,16-diene-epoxy-C15	46.40	17.90	60.02	29.50	46.90	1.89	1.54	40.60	62.20

P-glycoprotein inhibitor (Pgpi), P-glycoprotein substrate (Pgps), gastrointestinal absorption (GI-A), bioavailability (F30%), probability of crossing the blood-brain barrier (BBB), Half lifetime (T1/2), clearance rate (CL), hERG channel blocker (hERG), and human hepatotoxicity (H-HT).

REFERENCES

(1) Chakravarty, A. K.; Das, B.; Masuda, K.; Ageta, H. Tetracyclic triterpenoid*s* from *Glycosmis arborea. Phytochemistry.* **1996***, 42,* 1109-1113.

(2) El Dine, R. S.; El Halawany, A. M.; Nakamura, N.; Ma, C.-M.; Hattori, M. New lanostane triterpene lactones from the vietnamese mushroom *Ganoderma colossum* (FR.) C. F. BAKER. *Chem. Pharm. Bull.* **2008**, 56, 642–646.

(3) Hasegawa, S.; Miura, T.; Kaneko, N.; Hirose, Y.; Iitaka, Y. Further new rearranged lanostanoids from the seeds of Abies mariesii and A. firma. *Tetrahedron.* **1987***,* 43, 1775-1784.

(4) Tanaka, R.; Matsunaga, S. Triterpene lactones from the stem bark of *Abies firma. Phytochemistry.* ***1991****, 30,* 1983-1987.

(5) Cheng, C. R.; Yue, Q. X.; Wu, Z. Y.; Song, X. Y.; Tao, S. J.; Wu, X. H.; Guo, D. A. Cytotoxic triterpenoids from *Ganoderma lucidum. Phytochemistry.* **2010***, 71,* 1579-1585.

(6) Garlaschelli, L.; Vidari, G.; Virtuani, M.; Vita-Finzi, P.; Mellerio, G. The structures of new lanostane triterpenes from the Fruiting Boidies of *Hebeloma senescens. J. Nat. Prod.* **1995***, 58,* 992-1002.

(7) Luo, X. D.; Wu, S. H.; Ma, Y. B.; Wu, D. G. Tirucallane triterpenoids from *Dysoxylum hainanense. Phytochemistry.* **2000***, 54,* 801-805.

(8) Tanaka, R.; Matsunaga, S. 9β-Lanostane-Type triterpene lactones from the stem bark *of Abies veitchii. J. Nat. Prod.* **1991***, 54,* 1337-1344.

(9) Waddell, T. G.; Thomasson, M. H.; Moore, M. W.; White, H. W.; Swanson-Bean, D.; Green, M. E.; Fales, H. M . Isoflavone, wax and triterpene constituents of *Wyethia mollis. Phytochemistry.* **1982***, 21,* 1631-1633.

(10) Zhao, R.; He, Y. Network pharmacology analysis of the anti-cancer pharmacological mechanisms of *Ganoderma lucidum* extract with experimental support using Hepa1-6-bearing C57 BL/6 mice. *J. Ethnopharmacol*. **2018**, *210*, 287–295.

(11) Cherng, I. H.; Chiang, H. C.; Cheng, M. C.; Wang, Y. Three new triterpenoids from *Antrodia cinnamomea*. *J. Nat. P*rod. **1995**, *58*, 365-371.

(12) Ofodile, L. N.; Uma, N.; Grayer, R. J.; Ogundipe, O. T.; Simmonds, M. S. J . antibacterial compounds from the Mushroom *Ganoderma colossum* from Nigeria. *Phytother. Res.* **2011***,* 26*,* 748-751.

(13) Liu, J.; Kurashiki, K.; Shimizu, K.; Kondo, R. Structure–activity relationship for inhibition of 5α-reductase by triterpenoids isolated from *Ganoderma lucidum. Bioorg. Med. Chem.* **2006***, 14,* 8654-8660.

(14) Peng, X. R.; Wang, Q.; Su, H. G.; Zhou, L.; Xiong, W. Y.; Qiu, M. H. Anti-Adipogenic lanostane-type triterpenoids from the edible and medicinal mushroom *Ganoderma applanatum*. *J. Fungi*. **2022**, *8*, 331.

(15) Kikuchi, T.; Matsuda, S.; Kadota, S.; Murai, Y.; Ogita, Z. Ganoderic acid D, E, F, and H and lucidenic acid D, E, and F, new triterpenoids from *Ganoderma lucidum. Chem. pharm. bull.* **1985***,33,* 2624-2627.

(16) Peng, X.; Liu, J.; Xia, J.; Wang, C.; Li, X.; Deng, Y.; Qiu, M. Lanostane triterpenoids from *Ganoderma hainanense J. D. Zhao. Phytochemistry.* **2015***, 114,* 137-145.

(17) Arpha, K.; Phosri, C.; Suwannasai, N.; Mongkolthanaruk, W.; Sodngam, S. Astraodoric acids a-d: new lanostane triterpenes from edible mushroom *Astraeus odoratus* and their anti-mycobacterium tuberculosis h37ra and cytotoxic activity. *J. Agric. Food Chem.* **2012***, 60,* 9834-9841.

(18) Mendoza, G.; Suarez-Medellin, J.; Espinoza, C.; Ramos-Ligonio, A.; Fernandez, J. J.; Norte, M.; Trigos, A. Isolation and Characterization of Bioactive Metabolites from Fruiting Bodies and MYCelial Culture of *Ganoderma oerstedii* (Higher Basidiomycetes) from Mexico. Inter. J. Med Mushrooms. **2015**, 17, 501-509.

(19) Xia, Q.; Zhang, H.; Sun, X.; Zhao, H.; Wu, L.; Zhu, D.; She, G. A comprehensive review of the structure elucidation and biological activity of triterpenoids from *Ganoderma spp*. *Molecules.* **2014**, *19*, 17478-17535.

(20) Sato, H.; Nishitoba, T.; Shirasu, S.; Oda, K.; Sakamura, S. Ganoderiol A and B, new triterpenoids from the fungus *Ganoderma lucidum (Reishi). Agric. Biol. Chem.* **1986***, 50,* 2887-2890.

(21) Kitagawa, I.; Kobayashi, M.; Hori, M.; Kyogoku, Y. Marine natural products. XVIII. Four lanostane-type triterpene oligoglycosides, bivittosides A, B, C, and D, from the Okinawan sea cucumber *Bohadschia bivittata Mitsukuri. Chem. Pharm. Bull.* **1989***, 37,* 61-67.

(22) Akihisa, T.; Tagata, M.; Ukiya, M.; Tokuda, H.; Suzuki, T.; Kimura, Y. Oxygenated lanostane-type triterpenoids from the Fungus *Ganoderma lucidum. J. Nat. Prod.* **2005***, 68,* 559-563. doi:10.1021/np040230h

(23) Qiao, Y.; Zhang, X.; Qiu, M. *Two* novel lanostane triterpenoids from *Ganoderma Sinense. Molecules.* **2017***, 12,* 2038-2046.

(24) Ma, B. J.; Zhou, Y.; Ruan, Y.; Ma, J. C.; Ren, W.; Wen, C. N. Lanostane-type triterpenes from the sporoderm-broken spores of *Ganoderma lucidum. J. Antibiot.* **2012***, 65,* 165-167.

(25) Wei, J. C.; Wang, Y. X.; Dai, R.; Tian, X. G.; Sun, C. P.; Ma, X. C.; Wang, C. C27-Nor lanostane triterpenoids of the fungus *Ganoderma lucidum* and their inhibitory effects on acetylcholinesterase. *Phytochem. Lett.* **2017***, 20,* 263-268.

(26). Liu, L.Y.; Yan, Z.; Kang, J.; Chen, R. Y.; Yu, D. Q. Three new triterpenoids from *Ganoderma theaecolum. J. Asian Nat. Prod. Res.* **2017***, 19,* 847-853.

(27) Kleinwächter, P.; Anh, N.; Kiet, T. T.; Schlegel, B.; Dahse, H.-M.; Härtl, A.; Gräfe, U. *Colossolactones, New Triterpenoid Metabolites from a Vietnamese MushroomGanoderma colossum§. J. Nat. Prod.* **2001***, 64,* 236-239.

(28). Sappan, M.; Rachtawee, P.; Srichomthong, K.; Boonpratuang, T.; Choeyklin, R.; Feng, T.; Liu, J. K.; Isaka, M.; Ganoellipsic acids A-C, lanostane triterpenoids from artificially cultivated fruiting bodies of *Ganoderma ellipsoideum*. *Phytochem. Lett*. **2022**, 49, 27-31.

(29) Chairul, S. M.; Hayashi, Y. Lanostanoid triterpenes from *Ganoderma applanatum. Phytochemistry*. **1994***, 35,* 1305-1308.

(30) Fujita, A.; Arisawa, M.; Saga, M.; Hayashi, T.; Morita, N. Two new lanostanoids from *Ganoderma lucidum. J. Nat. Prod.* **1986***, 49,* 1122-1125.

(31) Liu, J. Q.; Wang, C. F.; Li, Y.; Luo, H. R.; Qiu, M. H. isolation and bioactivity evaluation of terpenoids from the medicinal fungus *Ganoderma sinense. Planta Med.* **2011***, 78,* 368-376.

(32) Zhang, W.; Tao, J.; Yang, X.; Yang, Z.; Zhang, L.; Liu, H.; Wu, J. Antiviral effects of two Ganoderma *lucidum triterpenoids* against enterovirus 71 infection. *Biochem. Biophys. Res. Commun.* **2014**, *449,* 307-312.

(33) Li, C.; Li, Y.; Sun, H. H. New ganoderic acids, bioactive triterpenoid metabolites from the mushroom *Ganoderma lucidum. Nat. Prod. Res.* **2006**, *20,* 985-991.

(34) Chairul, Tokuyama, T.; Hayashi, Y.; Nishizawa, M.; Tokuda, H.; Chairul, S. M.; Hayashi, Y. Applanoxidic acids A, B, C and D, biologically active tetracyclic triterpenes from *Ganoderma applanatum. Phytochemistry.* **1991**, *30,* 4105-4109.

(35) Nishitoba, T.; Sato, H.; Kasai, T.; Kawagishi, H.; Sakamura, S. New bitter C27and C30 terpenoids from the fungus *Ganoderma lucidum (Reishi). Agric. Biol. Chem.* **1984**, *48,* 2905-2907.

(36) Zamuner, M. L. M.; Cortez, D. A. G.; Dias Filho, B. P.; Lima, M. I. S.; Rodrigues-Filho, E. Lanostane triterpenes from the fungus *Pisolithus tinctorius. J. Braz. Chem. Soc.* **2005**, *16,* 863-867.

(37) Wang, K.; Bao, L.; Xiong, W.; Ma, K.; Han, J.; Wang, W.; Liu, H. Lanostane triterpenes from the Tibetan medicinal mushroom *Ganoderma leucocontextum* and their inhibitory effects on hmg-coa reductase and α-glucosidase. *J. Nat. Prod.* **2015**, *78,* 1977-1989.

(38) Isaka, M.; Chinthanom, P.; Rachtawee, P.; Choowong, W.; Choeyklin, R.; Thummarukcharoen, T. Lanostane triterpenoids from cultivated fruiting bodies of the wood-rot basidiomycete *Ganoderma casuarinicola. Phytochemistry.* **2020**, *170,* 112225.

(39) Gan, K. H.; Kuo, S. H.; Lin, C. N. Steroidal constituents of *Ganoderma applanatum* and *Ganodermaneo japonicum*. *J. Nat. Prod.* **1998**, *61*, 1421-1422.

(40) Kobori, M.; Yoshida, M.; Ohnishi-Kameyama, M.; Takei, T.; Shinmoto, H. (2006). 5α,8α-epidioxy-22e-ergosta-6,9(11),22-trien-3β-ol from an edible mushroom suppresses growth of hl60 leukemia and ht29 colon adenocarcinoma cells. *Bio. Pharm. Bull.* **2006**, *29,* 755-759.

(41) Han Y, Cheng Y, Tian LW. Semisynthesis of 22,25-epoxylanostane triterpenoids: structure revision and protective effects against oxygen-glucose deprivation/reoxygenation injury in H9c2 Cells. *J. Nat. Prod*. **2023**, *86*, 406-415.

(42) Li, Y. T.; Zhang, Z.; Feng, Y.; Cheng, Y.; Li, S.; Li, C.; Tian, L. W. Cardioprotective 22-hydroxylanostane triterpenoids from the fruiting bodies of *Phellinus igniarius. Phytochemistry*. **2021**, *191,* 112907.

(43) Min, B. S.; Nakamura, N.; Miyashiro, H.; Bae, K. W.; Hattori, M. Triterpenes from the spores of *Ganoderma lucidum* and their inhibitory activity against HIV-1 Protease. *Chem. Pharm. Bull.* **1998**, *46,* 1607-1612.

(44) El-Mekkawy, S.; Meselhy, M. R.; Nakamura, N.; Tezuka, Y.; Hattori, M.; Kakiuchi, N.; Otake, T. Anti-HIV-1 and anti-HIV-1-protease substances from *Ganoderma Lucidum*. *Phytochemistry.* **1998**, *49,* 1651-1657.

(45) Adams, M.; Christen, M.; Plitzko, I.; Zimmermann, S.; Brun, R.; Kaiser, M.; Hamburger, M. Antiplasmodial lanostanes from the *Ganoderma lucidum* mushroom. *J. Nat. Prod.* **2010**, *73,* 897-900.

(46) Akihisa, T.; Kokke, W. C. M. C.; Yokota, T.; Tamura, T.; Matsumoto, T. 4α,14α-dimethyl-5α-ergosta-7,9(11),24(28)-trien-3β-ol from *Phaseolus vulgaris* and *Gynostemma pentaphyllum. Phytochemistry.* **1990**, *29,* 1647-1651.

(47) Yue, J. M.; Chen, S. N.; Lin, Z. W.; Sun, H. D. Sterols from the fungus *Lactarium volemus. Phytochemistry.* **2001**, *56,* 801-806.

(48) Yang, S. X.; Yu, Z. C.; Lu, Q. Q.; Shi, W. Q.; Laatsch, H.; Gao, J. M. Toxic lanostane triterpenes from the basidiomycete *Ganoderma amboinense. Phytochem. Lett.* **2012**, *5,* 576-580.

(49) Guan, S. H.; Yang, M.; Liu, X.; Xia, J. M.; Wang, X. M.; Jin, H.; Guo, D. A. Two new lanostanoid triterpenes from the fruit body of *Ganoderma lucidum* the major component of SunRecome®. *Nat. Prod. Commun.* **2006**, *1*, 177-181.

(50) Nishitoba, T.; Goto, S.; Sato, H.; Sakamura, S. Bitter triterpenoids from the fungus *Ganoderma applanatum. Phytochemistry.* **1989**, *28,* 193-197.

(51) Hirotani, M.; Ino, C.; Furuya, T.; Shirot, M. Perenniporiol derivatives six triterpenoids from the cultured mycelia of *Perenniporia ochroleuca. Phytochemistry.* **1984**, *23,* 1129-1134.

(52) Fatmawati, S.; Shimizu, K.; Kondo, R. Ganoderic acid Df, a new triterpenoid with aldose reductase inhibitory activity from the fruiting body of *Ganoderma lucidum. Fitoterapia.* **2010**, *81,* 1033-1036.

(53) Yoshikawa, K.; Nishimura, N.; Bando, S.; Arihara, S.; Matsumura, E.; Katayama, S. New Lanostanoids, Elfvingic Acids A-H, from the Fruit Body of *Elfvingia applanata. J. Nat. Prod.* **2002**, *65,* 548-552.

(54) Yao, R.; Zhang, M.; Zhou, J. *et al.* Novel dual-targeting c-MYC inhibitor D347-2761 represses myeloma growth via blocking c-MYC/Max heterodimerization and disturbing its stability. *Cell Commun. Signal.* **2022**, *20*, 73.

(55) Yao, R.; Xie, Y.; Sun, X.; Zhang, M.; Zhou, J.; Liu, L.; Gao, J.; Xu, K. Identification of a Novel c-MYC Inhibitor 7594-0037 by Structure-Based Virtual Screening and Investigation of Its Anti-Cancer Effect on Multiple Myeloma. *Drug Des. Devel. Ther.* **2020***, 4,* 3983-3993.

(56) Singh, A.; Kumar, A.; Kumar, P.; Nayak, N.; Bhardwaj, T.; Giri, R.; Garg, N. A novel inhibitor L755507 efficiently blocks c-MYC-MAX heterodimerization and induces apoptosis in cancer cells. *J. Biol. Chem.* **2021**, *297*, 100903.

(57) Yap, J. L.; Wang, H.; Hu, A.; Chauhan, J.; Jung, K. Y.; Gharavi, R. B.; Prochownik, E. V.; Fletcher, S. Pharmacophore identification of c-Myc inhibitor 10074-G5. *Bioorg. Med. Chem. Lett*. **2013**, *23*, 370-374.
